# Genome-Wide Analysis of MicroRNA Responses to the Phytohormone Abscisic Acid in *Populus euphratica*

**DOI:** 10.3389/fpls.2016.01184

**Published:** 2016-08-17

**Authors:** Hui Duan, Xin Lu, Conglong Lian, Yi An, Xinli Xia, Weilun Yin

**Affiliations:** National Engineering Laboratory for Tree Breeding, College of Biological Sciences and Biotechnology, Beijing Forestry UniversityBeijing, China

**Keywords:** *Populus euphratica*, ABA, microRNA, high-throughput sequencing, target

## Abstract

MicroRNA (miRNA) is a type of non-coding small RNA with a regulatory function at the posttranscriptional level in plant growth development and in response to abiotic stress. Previous studies have not reported on miRNAs responses to the phytohormone abscisic acid (ABA) at a genome-wide level in *Populus euphratica*, a model tree for studying abiotic stress responses in woody plants. Here we analyzed the miRNA response to ABA at a genome-wide level in *P. euphratica* utilizing high-throughput sequencing. To systematically perform a genome-wide analysis of ABA-responsive miRNAs in *P. euphratica*, nine sRNA libraries derived from three groups (control, treated with ABA for 1 day and treated with ABA for 4 days) were constructed. Each group included three libraries from three individual plantlets as biological replicate. In total, 151 unique mature sequences belonging to 75 conserved miRNA families were identified, and 94 unique sequences were determined to be novel miRNAs, including 56 miRNAs with miRNA^*^ sequences. In all, 31 conserved miRNAs and 31 novel miRNAs response to ABA significantly differed among the groups. In addition, 4132 target genes were predicted for the conserved and novel miRNAs. Confirmed by real-time qPCR, expression changes of miRNAs were inversely correlated with the expression profiles of their putative targets. The *Populus* special or novel miRNA-target interactions were predicted might be involved in some biological process related stress tolerance. Our analysis provides a comprehensive view of how *P. euphratica* miRNA respond to ABA, and moreover, different temporal dynamics were observed in different ABA-treated libraries.

## Introduction

MicroRNA (miRNA), a kind of small non-coding RNA come from stem-loop structure, is single-stranded RNAs containing 18–24 nt (Bartel, 2004), that were first found in *Caenorhabditis elegans* lin-4 and let-7 (Lee et al., 1993; Reinhart et al., 2000). In plants, miRNAs were first reported in Arabidopsis in 2002 (Reinhart et al., 2002), and then identified in other plant species. miRBase (Release 21), the central database for miRNAs, lists 28,645 entries representing hairpin precursor miRNAs expressing 35,828 mature miRNA products in 223 species of plants, animals and viruse (Kozomara and Griffiths-Jones, 2014). MiRNA plays important roles in plant developmental processes and responses to biotic and abiotic stress (Kidner and Martienssen, 2005; Jones-Rhoades et al., 2006; Shukla et al., 2008; de Lima et al., 2012).

Gene expression can be regulated at transcriptional, posttranscriptional and translational levels, with miRNAs negatively regulating it at the posttranscription level (Obernosterer et al., 2006). With assistance from one of the argonaute proteins (AGO1), miRNAs regulate complementary target mRNA via specific cleavage or through translational inhibition (Phillips et al., 2007; Voinnet, 2009; Rogers and Chen, 2013). In plant, miRNAs are transcribed to form long primary transcripts (pri-miRNA). Then the pri-miRNAs are trimmed, producing miRNA precursors (pre-miRNA) with stem-loop secondary hairpin structure. Then pre-miRNA is cleaved into a double stranded RNA consisting of a miRNA and its complementary sequence miRNA^*^ (Park et al., 2002; Han et al., 2004; Kurihara et al., 2006). The double-stranded RNA is conveyed to the cytoplasm. One of the TWO strands acts as mature miRNA, loaded on the RNA induced silencing complex (RISC) to target mRNA (Bartel, 2004; Jones-Rhoades et al., 2006; Brodersen et al., 2008), meanwhile the other strand, miRNA^*^, is typically degraded (Jones-Rhoades et al., 2006).

The phytohormone abscisic acid (ABA) plays vital physiological roles in response to abiotic stress. The ABA level is controlled by complex regulatory mechanisms including biosynthesis, catabolism, transport and signal transduction pathways. The 9-cis-epoxycarotenoid dioxygenase 3 (NCED3), a key enzyme for ABA biosynthesis, is induced by drought stress, and it upregulates endogenous ABA levels in overexpressed transgenic plants (Tan et al., 1997; Thompson et al., 2000; Iuchi et al., 2001; Schwartz et al., 2003). CYP707A is the key enzyme for ABA catabolism. *Cyp707a3-1* mutant induces endogenous ABA level and reduces transpiration rate, thereby resulting in high tolerance to drought stress in Arabidopsis (Kushiro et al., 2004). Pyrabactin resistance1/PYR1-like/regulatory components of ABA receptors (PYR/PYL/RCAR protein family), the type 2C protein phosphatases (PP2Cs), and subfamily 2 of the SNF1-related kinases (SnRK2s) are a major breakthrough in the field of ABA signaling (Ma et al., 2009; Melcher et al., 2009; Nishimura et al., 2009; Park et al., 2009; Santiago et al., 2009; Yin et al., 2009; Gonzalez-Guzman et al., 2012). PP2C has a negative role in ABA signaling (Miyazono et al., 2009). Under stress conditions, the abundance of PP2C transcripts increase (Rubio et al., 2009; Szostkiewicz et al., 2010). With the help of ABA, SnRK2s are released from the PP2C inhibition, and activate their downstream targets (Klingler et al., 2010). *Srk2d srk2e srk2i (srk2d/e/i)* triple mutants show reduced tolerance to drought stress and highly enhanced insensitivity to ABA (Fujita et al., 2009). PYR/PYL/RCAR protein family, ABA receptor, play a major role in regulation of seed germination and establishment, basal ABA signaling required for vegetative and reproductive growth, stomatal aperture, and transcriptional response to the hormone(Gonzalez-Guzman et al., 2012).

Recent evidence indicates that miRNA and ABA affect each other, and that the expression levels of some miRNAs are regulated by ABA. For example, miR159, miR169, miR172 are regulated by ABA in the embryogenic callus of Japanese larch (*Larix leptolepis*) (Zhang et al., 2010). In maize roots, the entire miR169 family is downregulated by ABA (Luan et al., 2014). Under ABA treatment, the expression level of miR169a decreases, and the target of miR169, NFYA5 is upregulated (Li et al., 2008; Ni et al., 2013). The cistronic miRNA pair, miR842, and miR846, is a product of alternative splicing regulated by ABA in the roots of Arabidopsis. ABA regulating the alternative splicing, leading to reduce the expression of miR846, along with the accumulation of its target jacalin At5g28250 (Jia and Rock, 2013a,b).

Conversely, miRNAs change the sensitivity of plants to ABA. In Arabidopsis, miR160 overexpression reduces ABA sensitiveness during germination (Liu et al., 2007), and also causes abnormal root morphology, that leads to the lack of gravitropic responses in root tips and the promotion of more adventitious roots (Wang et al., 2005). In Arabidopsis, miR172b overexpression increased sensitivity to ABA and osmotic stress during a specific postgerminative stage (Zou et al., 2013).

In addition, exogenous ABA also influences some miRNA expression, with miRNA regulating the downstream genes of ABA. Overexpressing miR168a lead to ABA hypersensitivity and drought tolerance, while the loss-of-function mutant *miR168a-2* displays ABA hyposensitivity and drought hypersensitivity. Both the precursor and mature of miR168 are induced by ABA (Li et al., 2012). ABA also positively regulates both mature miR394 and precursor miR394a/b in Arabidopsis. Although ABA negatively regulates LCR, which is the target of miRNA394, the overexpression of miR394a/b leads to ABA hypersensitivity and ABA-associated phenotypes, whereas LCR overexpressing plants show ABA resistant phenotypes. Moreover, the overexpression of miR394a/b plants accumulate higher levels of ABA-induced hydrogen peroxide and superoxide anion radicals compared to wild-type and LCR-overexpressing plants (Song et al., 2013). MiR159 is induced by ABA, and it targeted MYB33 and MYB101 germinating seeds of Arabidopsis; the two MYB transcription factors are positively regulated by ABA. Overexpression of miR159a reduces sensitivity to ABA (Reyes and Chua, 2007). However, previous research has generally focus on the relationship between a specific single miRNA and ABA, with most regulation studied in the seed or root.

*Populus euphratica (P. euphratica)*, which exhibits remarkable tolerance to environmental stresses, is among a few tall tree that can survives in saline and alkaline area. It is a model woody plant for studying the molecular mechanisms of abiotic stress responses (Ye et al., 2009; Lv et al., 2014). Previous studies show miRNA participate in the drought and salt stress responses of *P. euphratica*, and the high-throughput sequencing has widely been used in miRNA research of *P. euphratica* (Li et al., 2011, 2013; Si et al., 2014), while other species poplus also involved in stress response (Ren et al., 2012, 2013, 2015; Chen et al., 2012a,b, 2015; Shuai et al., 2013). Hence, screening for ABA-responsive miRNA in *P. euphratica* may help elucidate the responses of woody plants to ABA and thus the mechanisms underlying such responses to abiotic stress; however, the relationship between the mechanism of ABA and miRNA regulation at a genomic level has not been reported. In this study, we attempt to provide new insight into this issue by identifying more relationships between ABA and miRNA regulation mechanisms in *P. euphratica* leaves.

## Materials and methods

### Plant material and growth conditions

Uniformly grown 1-year-old *P. euphratica* acquired from the Xinjiang Uygur Autonomous Region of China, were planted in individual 5 L pots containing a loam soil and placed in a greenhouse at Beijing Forestry University. Each container contained three individual plants of similar height. They were well watered and grown under natural conditions for 4 months (from April to July). The relative soil moisture content (RSMC) was measured using a FieldScout™ TDR 300 Soil Moisture Meter (Spectrum Technologies, Aurora, IL, USA). For all seedlings, the RSMC was controlled within 70–75%. To ensure that all leaves received a similar amount of ABA, an aqueous solution of ABA was used to water the *P. euphratica*. For the treated groups, 1 L 300 μM ABA solution was applied to each pot; and for the control group, pure water instead of ABA solution was used. Every pot was placed on a tray to prevent the solution from flowing away. Each group included three libraries from three individual plantlets as biological replicate. Leaves collected 1 day after adding the ABA solution were considered the short-term ABA treatment and named SL1, SL2, and SL3. Those collected 4 days after adding the ABA solution were considered the long-term ABA treatment and named LL1, LL2, and LL3. The control groups were named CL1, CL2, and CL3. Leaf tissues were collected at about 10:00 h, and all 8–12th (count from the apex) mature leaves, from independent plants in each group were collected. Prior to collecting, the photosynthetic rate, intercellular CO_2_ concentration, stomatal conductance and transpiration rate were measured using a Li-6400XT portable photosynthesis system (Li-Cor Inc., Lincoln, NE, USA). Two leaves in one plantlet were random selected, and measured with three technical repeat. And all three biological replicate for each group were measured. All of the collections were immediately frozen in liquid nitrogen, and stored at −80°C until RNA extraction.

### RNA extraction, libraries construction, and sequencing

Using the modified CTAB method, total RNA enriched with small RNA (sRNA) was isolated from the *P. euphratica* leaves in the nine collections (Jaakola et al., 2001). RNA quality and integrity were checked using a 2100 Bioanalyzer with the RNA 6000 Nano Kit (Agilent Technologies, Santa Clara, CA, USA). Sequencing libraries were generated using NEBNext® Multiplex Small RNA Library Prep Set for Illumina® (NEB, USA.) following manufacturer's recommendations. All the nine libraries from the three groups were for sRNA sequencing. High-throughput sequencing was performed using Illumina HiSeq technology was according to the manufacturer's protocol (Illumina, San Diego, CA, USA).

### Analysis of small RNA sequencing data

From the raw sequence reads obtained from the sRNA sequencing, we first removed low quality reads, including those shorter than 18 nt or longer than 30 nt, those with more than 10 nt single nucleotide repeats, or more than 10% N, and those with 5′ adapter contaminants, or without a 3′ adapter or the insert tag. Then the 3′ and 5′ adapters were removed to obtain clean reads without any mismatches, which were mapped to *P. euphratica* genome (Ma et al., 2013) using bowtie software (http://sourceforge.net/projects/bowtie-bio/files/) without any mismatch.

### sRNA reads annotation and miRNA identification

All mapped reads were annotated as follows. First, mapped reads were annotated as conserved miRNAs, which were previously discovered, which were registered in miRBase (Release 21) for *Populus trichocarpa* by BlastN algorithm with both mature and hairpin were without any mismatches. The remaining reads were annotated as non-coding RNA (i.e., tRNAs, rRNAs, scRNAs, snRNAs, and snoRNAs). The sequences were collected from the GenBank (http://www.ncbi.nlm.nih.gov/genbank/) and Rfam (11.0 release, http://rfam.xfam.org/) database. The similarity was investigated using the BlastN algorithm. The RepeatMasker was used to remove the repeat-associated RNAs (Repbase v.18.07, http://www.girinst.org/). Then nat-siRNAs were removed (*P. trichocarpa* in PlantNATsDB, http://bis.zju.edu.cn/pnatdb/). The remaining sRNA exactly matched the mRNA exons and introns in the *P. euphratica* genome (Ma et al., 2013). The remaining reads were used to predict novel miRNA utilizing miREvo and miRdeep2, based on secondary structure, the Dicer cleavage site, and the minimal folding free energies (Friedlander et al., 2012; Wen et al., 2012).

### Differential expression analysis of miRNA response to ABA

The expression levels of miRNA between the two groups were compared to determine which miRNAs were differentially expressed. The frequency of miRNA read counts was normalized as transcripts per million (TPM): normalized expression = (number of miRNA reads/total number of clean reads)^*^1,000,000 (Zhou et al., 2010). Raw data were used with the “DESeq2” library in the R statistical software package for this analysis (Love et al., 2014). The *P*-values were adjusted, and *P*-adjusted < 0.05 was considered to indicate significantly different expression (Benjamini and Hochberg, 1995).

### miRNA targets prediction and function analysis

Conserved and novel miRNA target were predicted by the psRNA Target Server (http://plantgrn.noble.org/psRNATarget/) with default parameters (Dai and Zhao, 2011). All discovered miRNA targets and differentially expressed miRNA targets were classified based on gene ontology (GO) performed using the online agriGO program (Du et al., 2010). miRNA targets prediction and GO classification analysis were based on the *P. euphratica* genome (Ma et al., 2013).

### Real-time quantitative PCR analysis of miRNAs and predicted targets

Ten miRNAs were randomly selected for real-time quantitative polymerase chain reaction (qPCR) for each comparison. The RNAs were extracted from each sample using CTAB method (Jaakola et al., 2001). The miRNA First-Strand cDNA Synthesis Kit (Aidlab Biotechnologies, Beijing, China), which is based on a poly-adenylation protocol was used for mature miRNA reverse transcription, and miRNA Real-Time PCR assay kit (Aidlab Biotechnologies, Beijing, China) was used for real-time qPCR. Real-time qPCR was performed using a total reaction volume of 20 μL, which contained 0.5 μL of diluted cDNA, 0.8 μM primer mix, 10.0 μL of 2 × miRNA qPCR Mix, and 8.7 μL ddH_2_O, which were performed using an ABI StepOnePlus™ instrument (Applied Biosystems, Foster City, CA, USA). Amplification reactions were performed as follows: 95°C for 10 s, 60°C for 20 s and 72°C for 30 s All reactions were performed in triplicate. *P. euphratica* 5.8 s rRNA was used as internal control for miRNA (Lu et al., 2008), and the 2^−ΔΔCT^ method was applied to calculate the relative changes in gene expression from real-time qPCR experiments (Livak and Schmittgen, 2001). All primers used for real-time qPCR are listed in Supplementary Data 7.

The expression analyses of several target genes were also examined using qRT-PCR. The FastQuant RT Kit (with gDNase) (Tiangen Biotech CO., LTD, Beijing, China), was used predicted targets mRNA reverse transcription, and SuperReal PreMix Plus (SYBR Green) (Tiangen Biotech CO., LTD, Beijing, China) was used for real-time qPCR according to the manufacturer's instructions. Real-time qPCR was performed using a total reaction volume of 20 μL, which contained 0.8 μL of diluted cDNA, 1.2 μM primer mix, 10.0 μL of 2 × SuperReal PreMix Plus, and 8.0 μL ddH_2_O, which were performed using an ABI StepOnePlus™ instrument (Applied Biosystems, Foster City, CA, USA). Amplification reactions were performed as follows: 95°C for 10 s, 60°C for 20 s and 72°C for 30 s. All reactions were performed in triplicate. *P. euphratica* UBQ was used as internal control for mRNA (Wang et al., 2015). And the 2^−ΔΔCT^ method was applied to calculate the relative changes in gene expression from real-time qPCR experiments (Livak and Schmittgen, 2001). All primers used for real-time qPCR are listed in Supplementary Data 7.

### Accession number

Sequencing data obtained in this work have been submitted to the Sequence Read Archive the accession number SRP077948.

## Results

### Physiological characterization of *P. euphratica* in response to ABA

**Figure 1:**
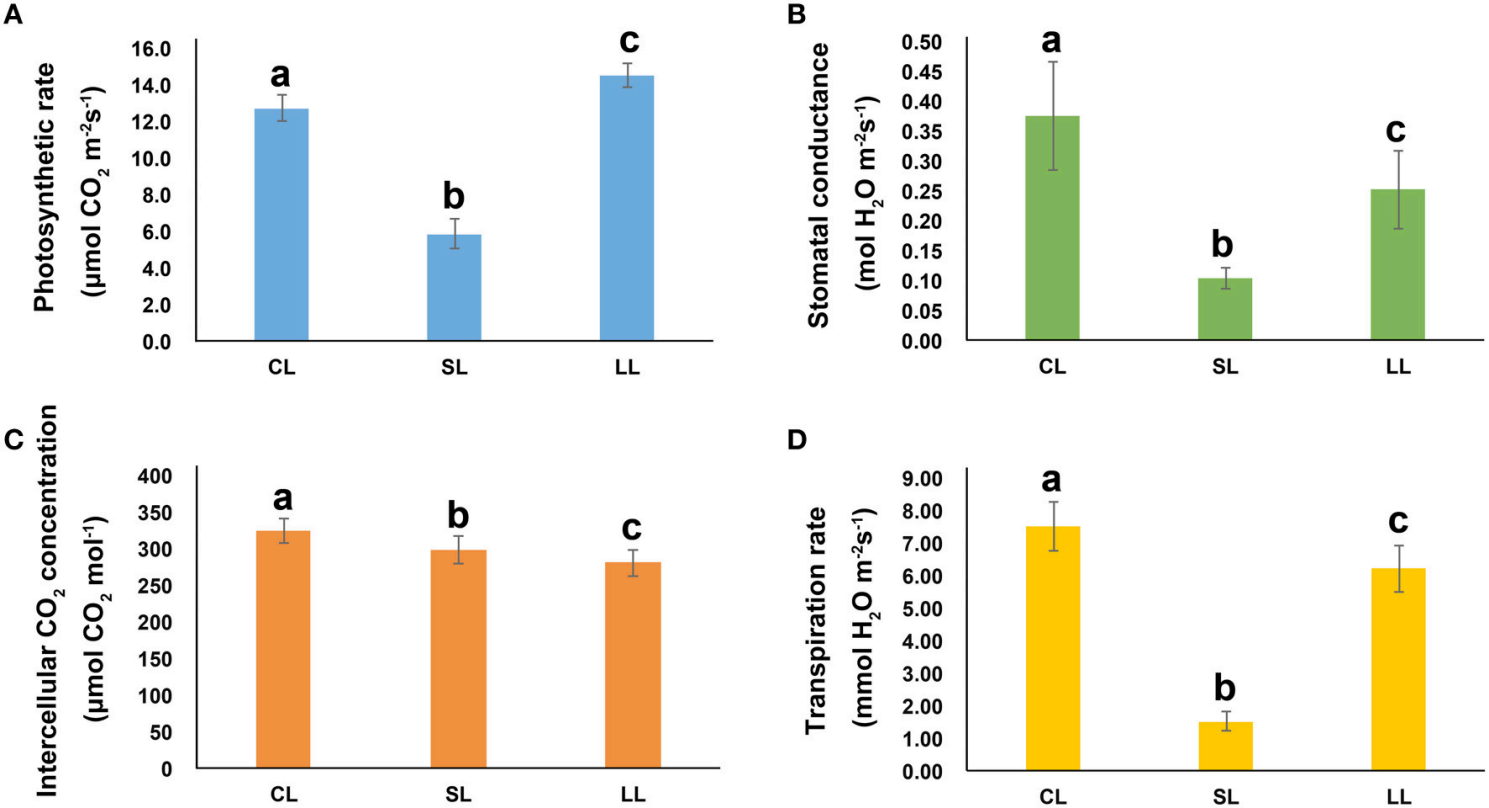
**The Response of *Populus euphratica* to ABA**. **(A)** Photosynthetic rate, **(B)** stomatal conductance, **(C)** intercellular CO_2_ concentration, and **(D)** transpiration rate of *P. euphratica* in response to ABA treatments for different amounts of time. Data are mean ± SE (*n* = 6). The values with different lowercase letters are significantly different at the *P* < 0.05 level.

One-year-old *P. euphratica* seedlings were exposed to soil with ABA. The photosynthetic rate, transpiration rate and stomatal conductance were greatly affected after 1 day of treatment, but had recovered by day 4. Compared to control, the photosynthetic rate decreased 55% on day 1 but was 1.1 times the control level on day 4 (Figure 1). Stomatal conductance decreased 80% after 1 day of treatment and recovered to 82% of the control level after 4 days of treatment. Similarly, the transpiration rate decreased 73% after 1 day of treatment, but recovered to 68% of the control level after 4 day of treatment. The intercellular CO_2_ concentration generally decreased over the 4 days compared to controls, and was 9 and 14% lower, respectively, after 1 and 4 days of treatment. In this study, the stomatal conductance decreased as the photosynthetic rate and transpiration rate fell after 1 day of treatment and then returned to normal, indicating that stomatal conductance was a key limitation to the photosynthetic rate in an early ABA response, although other factors, such as the activity of Rubisco, photosystem I (PSI), and photosystem II (PSII) were all essential factors for photosynthesis, may also inhibited photosynthesis under ABA conditions. The impact of the ABA treatment on photosynthesis in the early stage appeared to be recoverable.

### Overview of sRNA libraries data for high-throughput sequencing

To identify and characterize conserved and novel miRNAs in *P. euphratica*, nine sRNA libraries from CLs, SLs, and LLs were constructed. Through high-throughput sequencing, more than 10 million raw reads were obtained. After filtering the low-quality reads, and adaptor and contaminant sequences, ~90% of the clean reads remained (Table 1). Those unique sequences were then perfectly mapped to the *P. euphratica* genome (no mismatches allowed) (Ma et al., 2013), and the results showed that in most libraries over 50% of the total sRNA matched the *P. euphratica* genome perfectly. Among these libraries, nearly 60% of the total sequences were matched in all of the SL libraries, which was the highest proportion (Table 1).

**Table 1:** **Deep sequencing read statistics for nine small RNA libraries**.

	**CL libraries**			**SL libraries**			**LL libraries**		
Raw reads	12,029,234	12,029,235	12,029,236	12,029,235	12,029,236	12,029,237	12,029,236	12,029,237	12,029,238
Clean reads	11,467,477 (95.33%)	19,100,426 (96.21%)	11,189,160 (95.93%)	10,725,686 (95.44%)	11,100,606 (95.72%)	10,709,428 (95.95%)	10,725,686 (88.16%)	11,100,606 (97.36%)	10,709,428 (97.53%)
Length filtered unique reads	3,682,803	9,822,040	5,256,958	5,693,030	5,296,579	5,838,757	5,440,543	3,799,492	6,631,398
Mapped unique reads	2,427,929 (65.93%)	4,861,430 (49.50%)	2,986,411 (56.81%)	3,317,955 (58.28%)	3,200,469 (60.43%)	3,504,719 (60.03%)	3,148,652 (57.87%)	1,966,000 (51.74%)	3,553,027 (53.58%)
“+” Mapped unique reads	2,202,965 (59.82%)	4,212,799 (42.89%)	2,446,345 (46.54%)	2,784,750 (48.92%)	2,711,778 (51.20%)	3,036,658 (52.01%)	2,682,254 (49.30%)	1,628,852 (42.87%)	3,050,045 (45.99%)
“−” Mapped unique reads	224,964 (6.11%)	648,631 (6.60%)	540,066 (10.27%)	533,205 (9.37%)	488,691 (9.23%)	468,061 (8.02%)	466,398 (8.57%)	337,148 (8.87%)	502,982 (7.58%)

**Figure 2:**
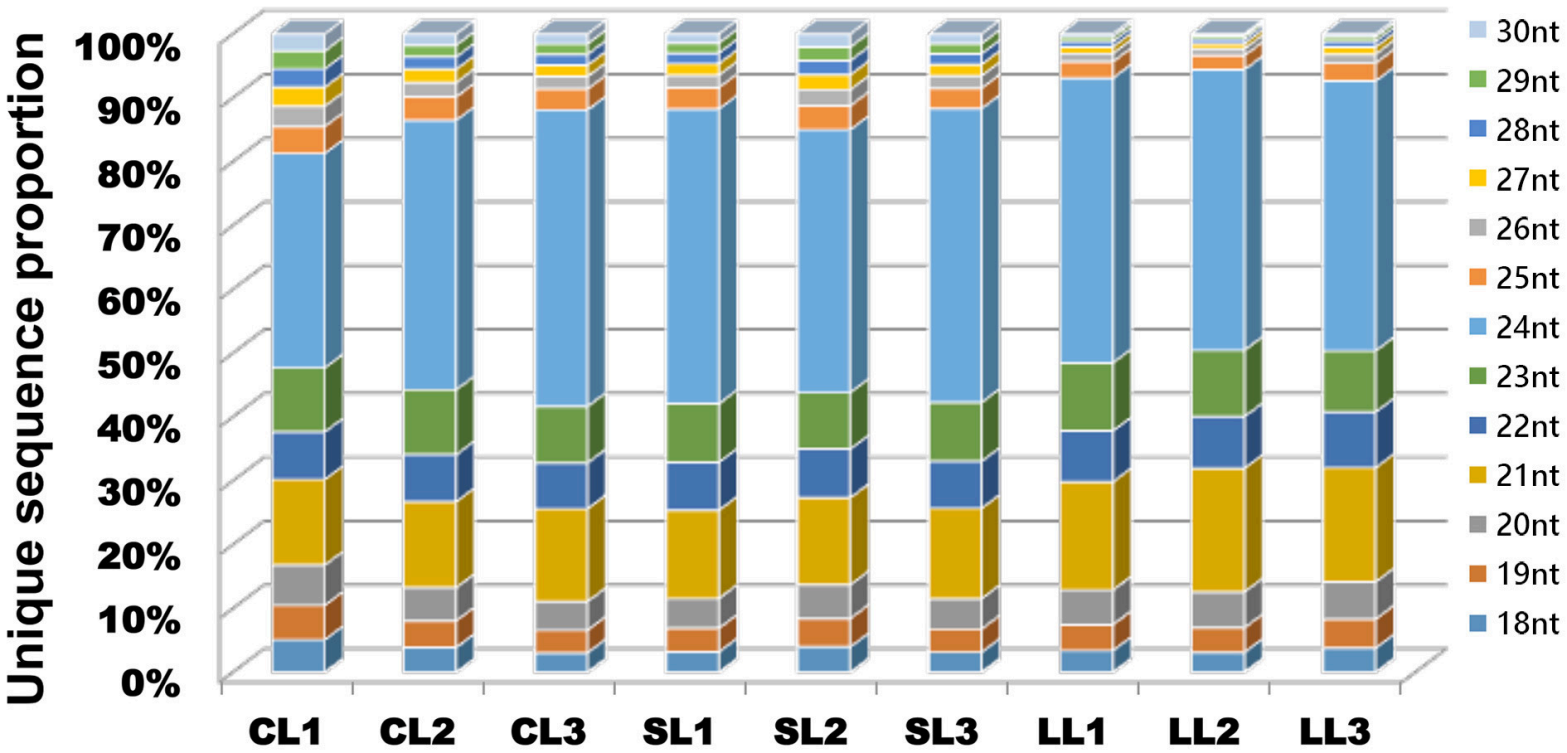
**Length distributions of small RNAs in nine samples**. Distribution of the sequence lengths of the sRNA from the nine libraries. Counts are based on unique sequences rather than the number of reads per unique sequence.

The size distribution of the sequencing of unique reads from the nine libraries was similar, and the majority of sRNAs were 21–24 nt in length (Figure 2). Specifically, 24 nt length reads were the most abundant in most libraries (except CL1), and over 40% were 24 nt sRNAs. The 21 nt class was the second most abundant. The sRNAs with different sizes perform different functions: 21 nt sRNAs usually mediate posttranscriptional gene silencing, while 24 nt sRNAs typically perform gene silencing mediated by RNA-dependent DNA methylation and heterochromatin maintenance (Zhang and Zhu, 2011; He et al., 2014; Lewsey et al., 2016).

### Conserved miRNAs discovered in *P. euphratica*

**Figure 3:**
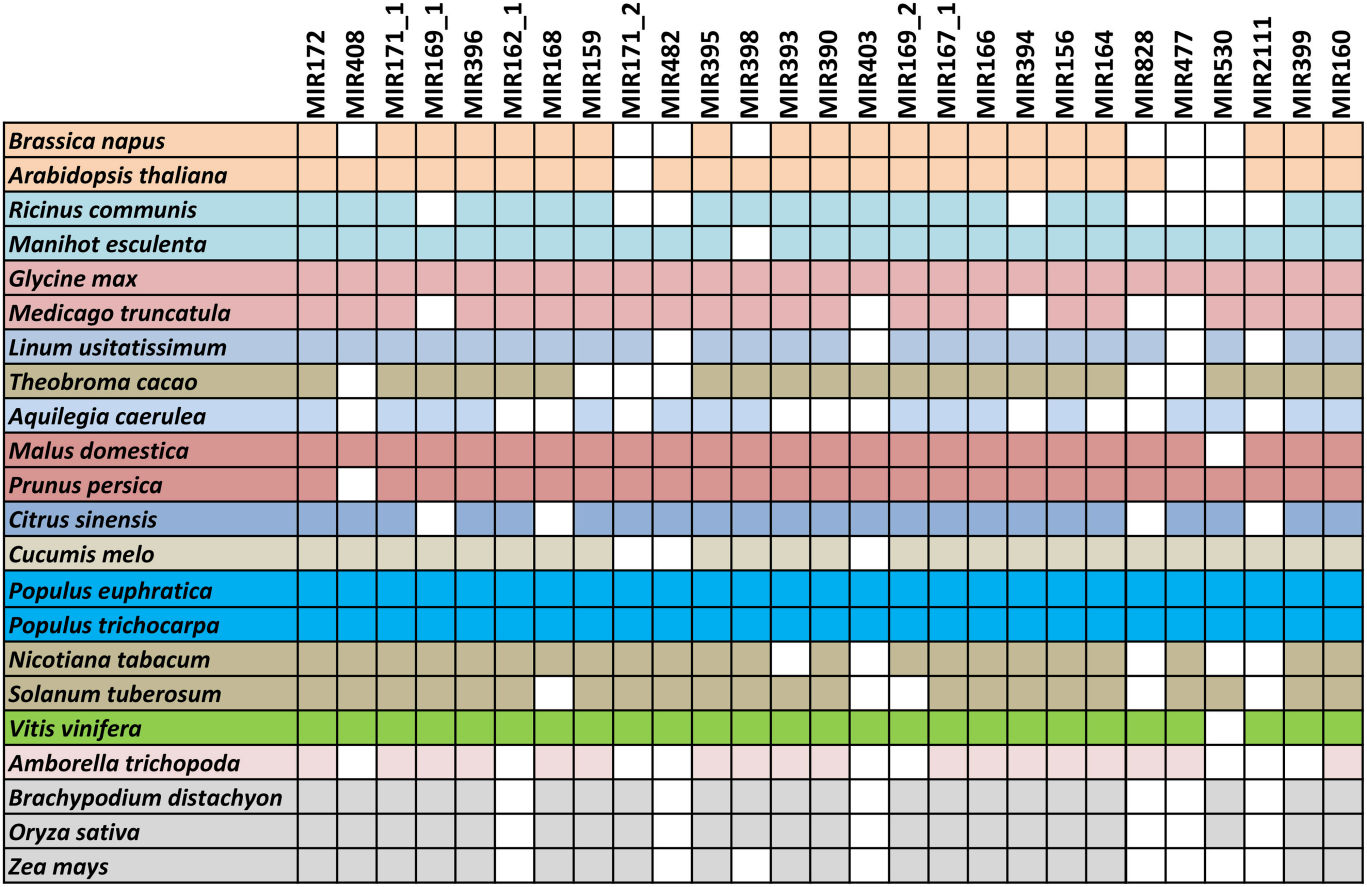
**Conserved miRNA families in *Populus euphratica* and across species**. Twenty-two representative conserved miRNA families in 23 angiosperms. All miRNAs of *P. euphratica* were identified based on sRNA sequencing data, and those of other plants were taken from miRBase (Release 21).

Conserved miRNA, non-coding RNA, repeat-associated RNA, nat-siRNA, exons, introns, and unknown sequences were identified successfully (Supplementary Data 1).The known conserved miRNAs were first identified, with no mismatches. Presently 253 unique mature sequences (include both 5p and3p), belonging to 136 families registered in miRBase (Release 21) for *P. trichocarpa*, and here 151 unique mature sequences, belonging to 75 miRNA families, were expressed in at least one of the nine libraries. Detailed information about the known miRNAs is shown in Supplementary Datas 2, 3. Seventy-two conserved miRNA families were identified, including 91 unique miRNAs belonging to 27 miRNA families and highly conserved (identified in more than 10 species of angiosperm, Figure 3), and 60 miRNAs belonging to 45 families were specific to Populus. The average TPM of the highly conserved and Populus-specific miRNA were 53,197.01 and 8,540.66, respectively. The abundance of highly conserved miRNA families was significantly higher than the Populus-specific miRNA.

### Analysis of novel miRNAs from *P. euphratica*

All novel miRNA candidates were obtained from miREvo and miRdeep2, and confidently met the novel miRNA requirements: (i) sequencings represented both the miRNA and miRNA^*^, and (ii) in miRNA^*^-deficient cases, the novel miRNA came from multiple and independent libraries(Xu et al., 2013). Ninety-four unique sequences were identified as novel miRNAs, while for 56 of them we found the corresponding miRNA^*^ sequences (Table 2 and Supplementary Data 4).

**Table 2:** **Novel miRNAs in *P. euphratica***.

	**TRS**	**Sequences (5′–3′)**	**Arm**	**LM (nt)**	**MEF (kcal/mol)**	**LP (nt)**	**GC%**	**MEFI**
miR-n1	8	gguaguucgaucguggaauu	5p	20	−57.8	244	0.34	0.69
miR-n2	564	gggaauguagccugacucgaga	5p	22	−38.4	84	0.50	0.91
miR-n3		uggugccacgcugcgugcgac	3p	21	−33.7	63	0.59	0.91
miR-n4	18	uuagucuuaauaauuguguga	5p	21	−44	80	0.19	2.93
miR-n5	24	gcaugaggggagucacgcagg	3p	21	−46.6	86	0.55	0.99
miR-n6		ucauuugagcaagaaauauu	3p	20	−52.2	129	0.34	1.19
miR-n7		gcggcagcaucaagauucaca	5p	21	−46.8	109	0.48	0.90
miR-n8		caggcggucuccuuggcua	5p	19	−78.9	215	0.47	0.78
miR-n9		cagaauugcagugccuugauc	3p	21	−41.4	98	0.40	1.06
miR-n10		gaaugcugaccgaauauggau	5p	21	−27.5	70	0.41	0.95
miR-n11		ucaauggccauuguaagagag	5p	21	−23.4	81	0.40	0.73
miR-n12		ucggaccaggcuucauccccc	3p	21	−30.4	84	0.50	0.72
miR-n13		guuccucugagcacaucacug	5p	21	−30.9	80	0.46	0.84
miR-n14		aagauggagaagcagggcacg	5p	21	−62.2	154	0.46	0.88
miR-n15	233	ugaugacugaucuugagcaug	5p	21	−41.1	81	0.49	1.03
miR-n16		gugggcuugugauccaagu	5p	19	−94.9	287	0.46	0.71
miR-n17		cuuggcuauuguagauaaccc	3p	21	−39.5	93	0.46	0.92
miR-n18	122	ggauugucgucugguucgaug	5p	21	−33.2	83	0.41	0.98
miR-n19		ucagcgcugcauucaaucaug	3p	21	−39.7	104	0.42	0.90
miR-n20	5	cucaagaaagccgugggaga	3p	20	−42.3	127	0.42	0.80
miR-n21		uaucccuuuggauuccuccuu	3p	21	−43.8	82	0.51	1.04
miR-n22		guuccuccuagcuucuucagug	5p	22	−61	207	0.40	0.74
miR-n23		guuccucugagcacuucaacg	5p	21	−33.1	80	0.46	0.89
miR-n24	7	cucccccucaagggcuuccugu	5p	22	−54.9	110	0.51	0.98
miR-n25	14	ucggaccaggcuucauuccuc	3p	21	−25.7	82	0.46	0.68
miR-n26	53	aguuuguucguggaacugaug	5p	21	−28.6	80	0.48	0.75
miR-n27	14	ucgagaucuguucaauaguac	5p	21	−89.9	248	0.33	1.11
miR-n28	6	uauuggccugguucacucaga	5p	21	−36.4	106	0.40	0.87
miR-n29	385	aggugcaggugcuagugcagg	3p	21	−51.3	151	0.39	0.87
miR-n30	564	uaaugcugucugguucgagac	5p	21	−35.3	80	0.50	0.88
miR-n31	160	uugacagaagagagugagcac	5p	21	−42	85	0.38	1.31
miR-n32	14	cucggaccaggcuucauuccc	3p	21	−36.9	73	0.52	0.97
miR-n33	1	uuucgggaagugaaauuugga	3p	21	−35.8	113	0.36	0.87
miR-n34	26	ggucaugcucugacagccucacu	3p	23	−31	89	0.45	0.78
miR-n35	2	ugagaucuuuugaauuauaucauu	5p	24	−58.9	233	0.28	0.89
miR-n36	74	auugaugguagaauuacuugg	3p	21	−32	78	0.28	1.45
miR-n37	102	auaaagugggcaguagagagu	5p	21	−48.3	78	0.40	1.56
miR-n38	26	caacccuuccagauauuggug	3p	21	−47.3	61	0.52	1.48
miR-n39	3	uggacugaagggagcuccuuc	3p	21	−79.2	184	0.43	0.99
miR-n40	108	uucauguaucucucgacucuu	3p	21	−39.9	93	0.34	1.25
miR-n41	130	auuugcuuguauuuaacuccc	5p	21	−51.1	75	0.43	1.60
miR-n42	1	ugacaggcucuucucucucaug	3p	22	−64.8	172	0.41	0.93
miR-n43	1	gaauguugucuggcucgagga	5p	21	−42.6	110	0.41	0.95
miR-n44	15	auaugcguuuuugucccucgc	3p	21	−83.8	264	0.42	0.75
miR-n45		cacgugcuccccuucuccaac	3p	21	−34.9	66	0.55	0.97
miR-n46		caugugcucuagcucuccagc	3p	21	−41.7	74	0.47	1.19
miR-n47	57	uauuauaaccaagacuaaugc	5p	21	−37.7	120	0.24	1.30
miR-n48	2709	uuacacuggcaacucauuuga	3p	21	−62.6	186	0.37	0.92
miR-n49	38	cgggguuggguguucuaugag	3p	21	−42.3	85	0.49	1.01
miR-n50	1	uuuaugcguuuuuggcccucg	3p	21	−36.7	119	0.39	0.80
miR-n51	331	acacacacuggagaagggaag	3p	21	−47.2	71	0.56	1.18
miR-n52	1	aaguaccugauagcaacucaagu	5p	23	−84.4	278	0.46	0.66
miR-n53	1	gaagauuuggugugccucugaggu	3p	24	−68.6	226	0.39	0.78
miR-n54		ugccaaaggagaguugcccug	3p	21	−40.2	87	0.49	0.93
miR-n55	53	uuguaaugauauagaugugau	5p	21	−43.2	103	0.26	1.60
miR-n56	4	ucaauggagcccagauguaga	5p	21	−33.4	78	0.36	1.19
miR-n57	2	uugaauaugcuugagcugugu	5p	21	−36	64	0.36	1.57
miR-n58	1	ugaaaagacgaaagaguagaga	5p	22	−63.2	140	0.44	1.02
miR-n59	1	aggggcaaaaaucgcauaaga	5p	21	−43.9	105	0.43	0.98
miR-n60	3	uuaagucgcaugcaugauuga	3p	21	−68.9	234	0.38	0.77
miR-n61	1	uguugaugaugauaaauu	5p	18	−64.3	292	0.21	1.04
miR-n62	1	uagccaaggaugacuugcccgc	5p	22	−50.7	122	0.44	0.94
miR-n63	21	uuauaaagccauaagaagucc	5p	21	−47.3	76	0.26	2.37
miR-n64	11	aagagacucuuguauguauguaug	5p	24	−41.1	59	0.31	2.28
miR-n65	795	uucugucgccggaaagauggug	5p	22	−43.7	82	0.48	1.12
miR-n66	4	agaaagaagacagaaggcagcgug	5p	24	−67.8	105	0.44	1.47
miR-n67	13	acauaaauugcaaggaacuua	5p	21	−30.2	72	0.31	1.37
miR-n68		gugaggcugguuucacagagc	3p	21	−41.6	115	0.50	0.72
miR-n69	90	cucgaugaccgaucuugggca	5p	21	−38.3	84	0.49	0.93
miR-n70		guuccccugagcacuucacu	5p	20	−42.1	82	0.44	1.17
miR-n71		auuagauguagguuugauacgugu	3p	24	−31	166	0.23	0.79
miR-n72	6	uuucaguuugauucagcaugc	5p	21	−49.7	193	0.36	0.72
miR-n73		auaaacuugauuauguggaauaua	5p	24	−127	254	0.25	2.02
miR-n74	183	ugaacucucucccucaacugc	5p	21	−41.5	85	0.48	1.01
miR-n75	10	uuuguaauuuguaaacuuguu	3p	21	−73.3	123	0.29	2.04
miR-n76		ugaggccuuugggggagaguga	3p	22	−40.7	89	0.53	0.87
miR-n77		gcgaccccaggucaggcgga	5p	20	−98.2	220	0.64	0.70
miR-n78	1	uuuuucuugguguuguuggacucu	3p	24	−70.1	148	0.41	1.17
miR-n79		uaagaucauugccuauuggagacu	3p	24	−29.2	81	0.37	0.97
miR-n80		ugcucacuucucuucugucagc	3p	22	−49.2	85	0.46	1.26
miR-n81		uugacagaagauagagagcacu	5p	22	−41.2	87	0.41	1.14
miR-n82		uuuggaaggaagauuugaagu	3p	21	−61.9	180	0.42	0.81
miR-n83		auuuaaauugauucugaaacuauu	3p	24	−88.9	221	0.17	2.34
miR-n84		uuggacugaagggagcucccuc	3p	22	−80.4	179	0.46	0.97
miR-n85		aggugcuggugccggugcagg	3p	21	−40.3	112	0.40	0.90
miR-n86		ccucgcucccagcugacaccc	5p	21	−73.7	195	0.49	0.77
miR-n87		aaauugaugaauuuauggagu	3p	21	−47.2	155	0.40	0.76
miR-n88	24	gugcucacgucucuucugucag	3p	22	−53.3	85	0.47	1.33
miR-n89	4010	uuccaaagggaucgcauugauc	5p	22	−42.5	93	0.37	1.25
miR-n90		uaugggaggauuggacaggac	5p	21	−34.1	75	0.48	0.95
miR-n91		uagccaaggacgauuugccugu	5p	22	−47.7	99	0.53	0.92
miR-n92	93	aaccccuaguugcacguggacgug	5p	24	−49.6	109	0.59	0.78
miR-n93		uucgaucugggucaaaucuuuc	3p	22	−30.4	56	0.52	1.05
miR-n94		ggagcgaccuggaaucacaug	5p	21	−38.3	76	0.61	0.83

TSR, total read count of miRNA^*^; nt, nucleotide; LM, length of miRNA; LP, length of precursors.

**Figure 4:**
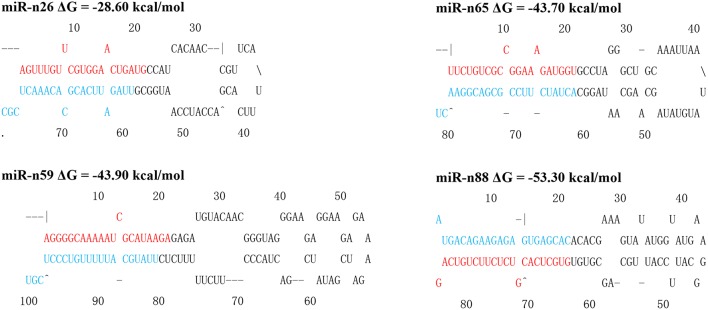
**Predicted miRNA precursor stem–loop structures of novel miRNA precursors**. Precursor structures of four novel *P. euphratica* miRNAs (miR-n26, miR-n59, miR-n65, and miR-n88) were predicted by the online software MFOLD. The MEFs are shown after the miRNA names. The mature miRNA and miRNA star sequences are marked in red and blue, respectively.

The length of mature novel miRNA sequences were 18–24 nt. Mfold was utilized to predict the stem-loop structure of precursors of all non-conserved miRNAs (Zuker, 2003), and the information was provided in Figure 4 and Supplementary Figure 1. The pre-miRNA length ranged from 56 to 292 nt. The negative minimal folding free energies (MFEs) varied from −127.0 to −23.4 kcal/mol; the average value was –49.47, which was much less than that of the tRNA (−27.5 kcal/mol) and rRNA (−33 kcal/mol) (Bonnet et al., 2004). The minimal folding free energy index (MFEI) values ranging from 0.66 to 2.93. Seventy-one miRNAs (~74%) were above 0.85, which was a key characteristic in distinguishing pre-miRNAs from other sRNAs (Zhang et al., 2006). The MFEI of all pre-miRNAs was significantly higher than that of tRNAs (0.64), rRNAs (0.59), and mRNAs (0.62–0.66). All data indicated that pre-miRNAs possessed highly stable hairpin structures. A nucleotide bias tendency indicated that in 46 of 94 cases, the first nucleotide was U in 48% of the novel non-conserved miRNAs, in agreement with that of conserved miRNAs in other plants (Voinnet, 2009). These results agree with that miRNAs were preferentially loaded onto AGO1-containing RISC, and preferentially contain a U at the 5′-end (Table 2; Mi et al., 2008).

The expression levels of these novel miRNA showed a large TPM range (Supplementary Data 3). Seven of 94 novel miRNA were above 10,000 TPM for the sum of all nine libraries, with the most abundant being miR-n1 at an amazing 623,863.3 TPM. More than half of these miRNAs (~78%) were discovered to be less than 1000 TPM, which is consistent with the finding that novel miRNAs are often expressed at a lower level compared to conserved miRNAs.

The average TPM of all Populus-specific microRNAs, including known Populus-specific microRNAs and novel miRNA was 8,611.81. The abundances of those consistent with species-specific conserved miRNAs were expressed lower than those of highly conserved miRNAs (Zhao et al., 2010).

### Differentially expressed miRNAs response to ABA

To compare the expression of miRNA in different samples, the “DEseq2” library in the R statistical software package was performed on the raw counts for all of the conserved and novel miRNAs. The analysis of the differential expression of miRNAs showed that 31 known miRNAs and 31 novel miRNAs under ABA were significantly differentially expressed in *P. euphratica* (Table 3 and Supplementary Data 5).

**Table 3:** **Significantly differentially expressed miRNA showing log2-fold changes in expression**.

**miRNA**	**SL vs. CL**		**LL vs. CL**		**LL vs. SL**	
	**Log2-fold change**	**Significant level**	**Log2-fold change**	**Significant level**	**Log2-fold change**	**Significant level**
peu-miR1446	1.05	^*^	1.17	^*^		
peu-miR1448					−0.32	^**^
peu-miR156a			0.79	^**^	1.05	^**^
peu-miR160a-3p			−1.17	^*^		
peu-miR160b-5p			0.91	^*^	0.65	^*^
peu-miR164a	1.10	^**^	1.82	^**^	0.66	^*^
peu-miR167b					0.26	^*^
peu-miR167c-3p	−0.90	^*^			0.76	^**^
peu-miR167c-5p					0.40	^**^
peu-miR168-3p	−0.67	^**^	−0.73	^**^		
peu-miR168-5p	−1.30	^*^				
peu-miR171c-3p			−1.15	^*^	−1.05	^**^
peu-miR171d-5p			−1.68	^*^		
peu-miR319a	0.70	^**^			−0.62	^**^
peu-miR319b	0.58	^*^			−0.83	^**^
peu-miR395a	1.27	^*^	2.96	^**^	1.58	^**^
peu-miR395b			2.07	^**^	1.82	^**^
peu-miR398b	0.95	^**^			−0.50	^**^
peu-miR399c			1.50	^**^	1.65	^**^
peu-miR403-3p					−0.93	^**^
peu-miR408-3p	0.88	^**^			−0.61	^*^
peu-miR408-5p	0.99	^*^				
peu-miR472a					−0.78	^**^
peu-miR472b					−0.47	^*^
peu-miR475a-5p			0.84	^*^	1.60	^**^
peu-miR477a-3p					1.06	^**^
peu-miR482a.2			−0.76	^**^	−0.78	^**^
peu-miR6421	−2.10	^**^	1.28	^**^	3.56	^**^
peu-miR6462a-3p			−2.04	^**^	−1.72	^**^
peu-miR6462b	−1.69	^**^	−2.33	^**^		
peu-miR7814					−0.92	^*^
peu-miR-n2	−1.57	^**^	−2.21	^**^	−0.66	^**^
peu-miR-n10					−1.17	^**^
peu-miR-n11	1.19	^*^	1.61	^**^		
peu-miR-n13			2.24	^**^	0.83	^*^
peu-miR-n19	1.90	^**^			−2.63	^**^
peu-miR-n24	−1.20	^*^				
peu-miR-n25	0.77	^**^				
peu-miR-n26			−1.35	^**^	−0.83	^*^
peu-miR-n29					0.88	^**^
peu-miR-n30					1.34	^*^
peu-miR-n31					1.23	^**^
peu-miR-n32	−1.28	^**^	−1.93	^**^		
peu-miR-n34			−1.97	^**^	−1.64	^**^
peu-miR-n35			−1.43	^*^		
peu-miR-n37			2.27	^**^		
peu-miR-n38					−1.40	^*^
peu-miR-n49					1.52	^**^
peu-miR-n51			1.50	^*^	1.98	^**^
peu-miR-n58	1.61	^**^	1.77	^**^		
peu-miR-n59	−1.63	^**^	−2.02	^**^		
peu-miR-n62					−1.71	^**^
peu-miR-n65	−1.81	^**^	−1.34	^**^		
peu-miR-n74					0.57	^**^
peu-miR-n75					−0.95	^*^
peu-miR-n77			−1.49	^*^	−1.19	^*^
peu-miR-n81	−0.70	^*^			0.96	^**^
peu-miR-n86					1.41	^*^
peu-miR-n87	−2.03	^**^	−2.82	^**^		
peu-miR-n88	−1.25	^**^			1.28	^**^
peu-miR-n91	3.68	^**^	3.46	^**^	−0.43	^*^
peu-miR-n94	0.82	^*^			−1.19	^**^

* *P < 0.05*,

** *P < 0.01*.

*Red means miRNA up-regulated by ABA, and blue means down-regulated by ABA. The darker color means the greater the change, and vice versa in one comparison*.

Results showed that of miRNA expressed after 1 day of treatment, eight conserved and six novel miRNAs were upregulated, whereas five conserved and eight non-conserved miRNA were downregulated, compared to that control. Among them miR-n91 and peu-miR408-3p were upregulated, and peu-miR6421 and miR-n87 was downregulated. Similarly, after 4 days of treatment, eight conserved and six novel miRNAs were upregulated, whereas seven conserved and nine novel miRNAs were downregulated, compared to the control. For example, miR-n87 and peu-miR6462-3p were downregulated, and peu-miR6421 and miR-n9 were upregulated. After 4 days of treatment, 11 conserved and 10 novel miRNAs were upregulated, whereas 12 conserved and 11 novel miRNAs were downregulated, compared to those after 1 day of treatment. For example, miR-n19 was downregulated, and peu-miR6462-3p and peu-miR6421 were down-regulated.

In comparing the three groups with each other, only five miRNAs (miRNA-n2, miRNA-n91, peu-miR164a, peu-miR395a, and peu-miR6421) were all significantly differentially expressed. Compared to the control libraries, nine miRNAs were significantly differentially expressed in both the 1- and 4-day ABA treatment libraries. Compared to the control, the expression levels of three miRNAs were all significantly increased in the 1 day of treatment libraries, and the other six miRNAs decreased for 4 days. Between SL vs. CL and LL vs. SL, nine miRNAs were significantly differentially expressed. Three of them first ascended, then descended, while the others were opposite. Compared to the 4 days of treatment libraries, both in the control and 1 day of treatment libraries, 11 miRNAs were significantly differentially expressed. Five of them were upregulated, and other six miRNAs were all downregulated.

### Confirmation of miRNAs by real-time-qPCR

**Figure 5:**
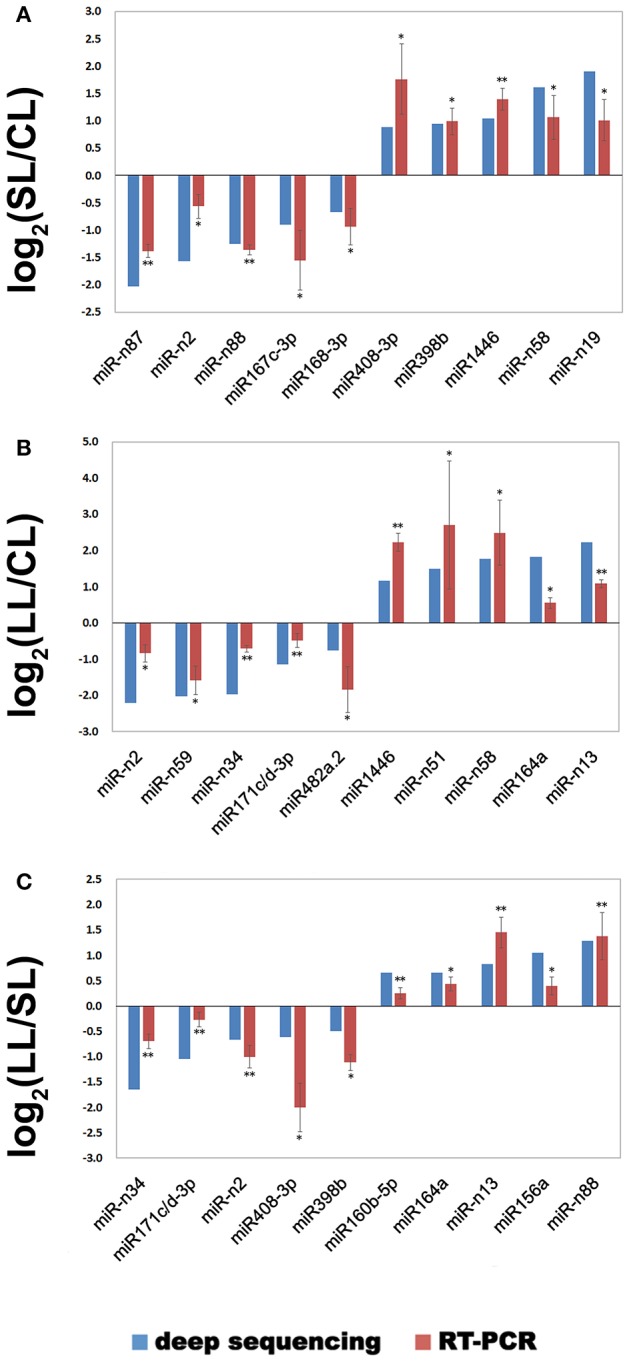
**Verification of selected miRNAs by real-time quantitative PCR**. Differentially expressed miRNAs identified by sequencing were confirmed by real-time qPCR, and their expression levels were compared among the three groups. The expression level of miRNA in deep sequences was performed with R statistical software package, which was“DESeq2” library used with raw date. ^*^P < 0.05, ^**^P < 0.01. **(A)** The miRNA expression for the comparison SL/CL; **(B)** The miRNA expression for the comparison LL/CL; **(C)** The miRNA expression for the comparison LL/SL.

Real-time qPCR analysis was utilized to confirm the expression patterns for the significantly differentially expressed miRNA. Ten miRNAs were selected for each comparison (SL/CL, LL/CL, and LL/SL) with three experimental and three biological replicates to validate and measure the sequencing results. The comparison expression levels of these miRNAs between real-time qPCR and sequencing analyses were consistent (Figure 5).

### Prediction and annotation of target genes of miRNAs

To understand the functions of miRNA responses to ABA, the prediction and identification the function of their targets is a crucial step of in analysis. The psRNA Target Server was used to predicted target of miRNAs. In total of 4132 targets genes were predicted for known and novel miRNAs. 1584 were for novel miRNAs, and 2548 were for conserved miRNAs (Supplementary Data 6).

Generally, several targets were regulated by a single miRNA. MiRNA-n61 targeted 155 transcripts, the most targets of all discovered miRNA. Considering conserved miRNAs only, miRNA482a.1 target 129 transcripts. Whereas a single gene could be targeted by multiple miRNA. CCG015515.1 was targeted by 12 members of the miRNA169 family and two novel miRNAs, miR-n62 and miR-n91. Furthermore, one gene being targeted by several miRNAs from at least two miRNA families was not unusual. In particular, CCG030854.1 was targeted by six miRNAs, peu-miR478b, peu-miR478d, peu-miR481a, peu-miR481b, peu-miR6421, and peu-miR7812, which were belonged to four different miRNA famines.

**Figure 6:**
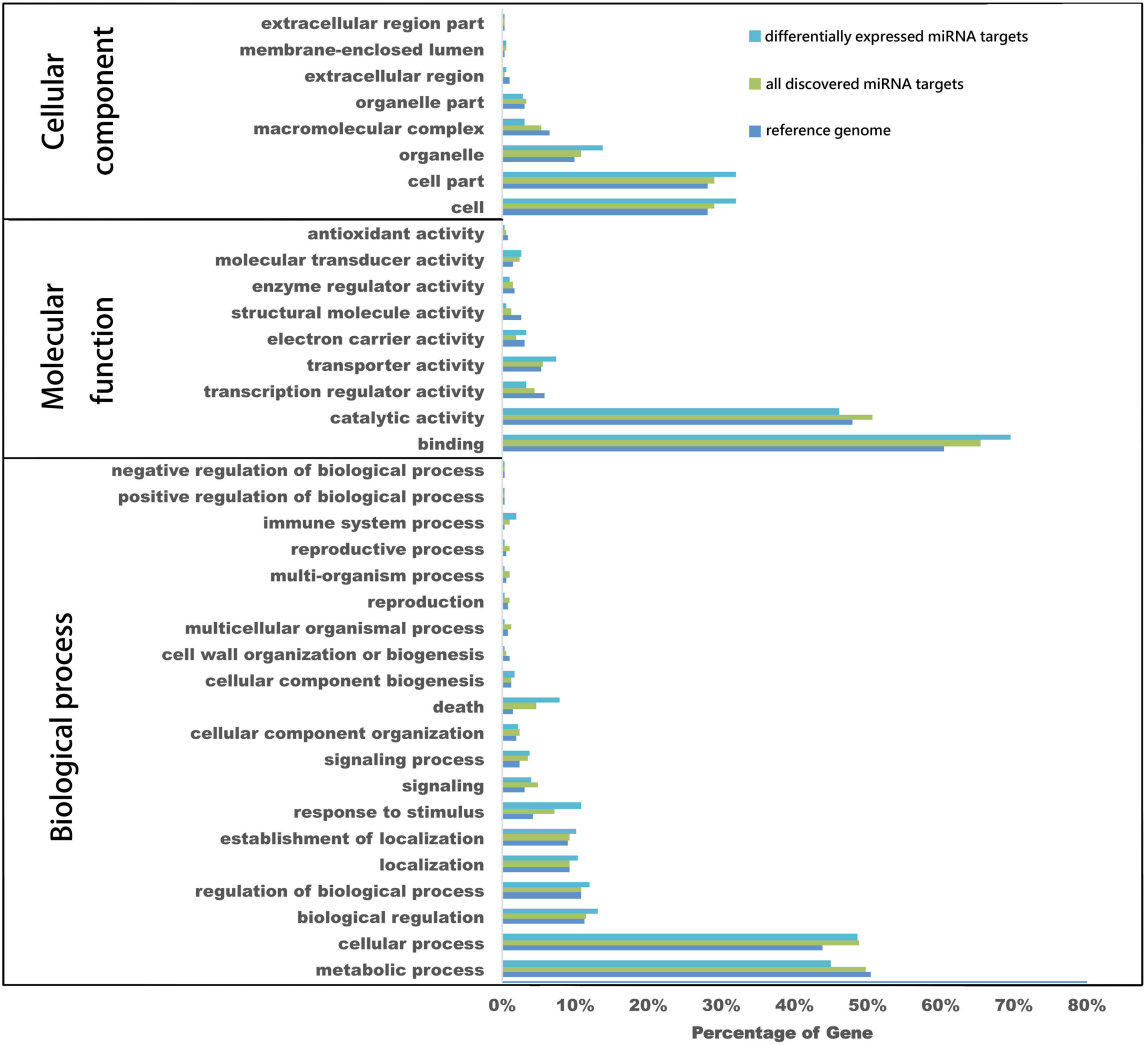
**GO analysis of miRNA putative target genes**. GO annotation categorized all of the predicted miRNA target genes and differentially expressed miRNA target genes into biological processes, molecular functions, and cellular components.

To better understand the functions of these genes, GO analysis was employed to classify target genes based on their involvement in biological processes, cellular components and molecular functions. Findings showed that 2179 of all predicted target genes could be categorized into totally 706 GO terms, including 260 biological processes, 365 molecular functions and 81 cellular components. For all differential expressed miRNA targets 280 GO terms, included 139 biological processes, 116 molecular functions and 25 cellular components. Compared to all discovered miRNA targets, some GO terms had different proportions. The secondary level GO terms for all of differentially expressed miRNA targets, all miRNA targets and the reference genome was determined (Figure 6). In this study, the cellular components, the proportions of differentially expressed miRNA targets of “organelle” (GO: 0043226), “cell part” (GO: 0044464), and “cell” (GO: 0005623) were higher compared to those of all discovered miRNAs. In molecular functions, that of “binding” (GO: 0005488), “transporter activity” (GO: 0005215) and “electron carrier activity” (GO: 0009055) were also higher than that for all discovered miRNA here. Similarly, in biological processes, those of “death” (GO: 0016265), “response to stimulus” (GO: 0050896), and “immune system process” (GO: 0002376) were higher than that for all of the discovered miRNAs.

### Expression analyses of predicted target genes

**Figure 7:**
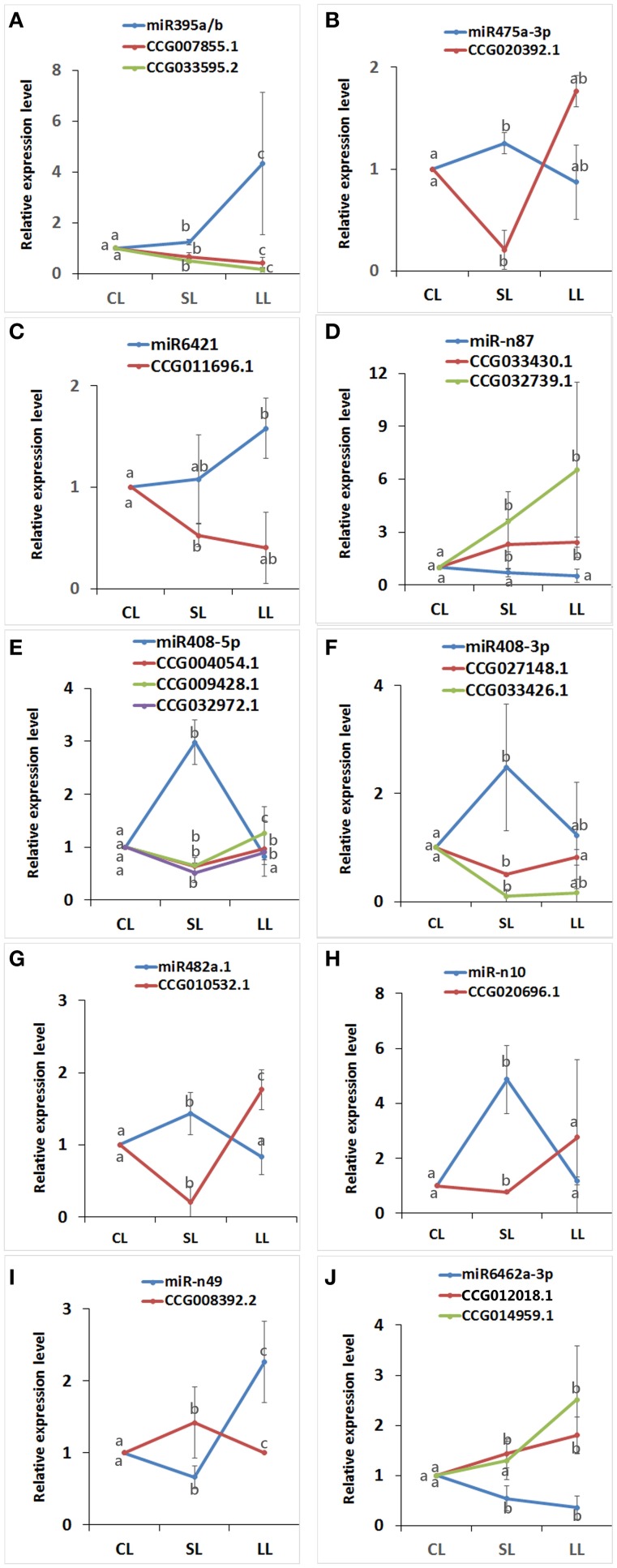
**The expression profiles of predicted target genes and their corresponding miRNAs by real-time quantitative PCR**. Complementary miRNAs and predicted target gene were confirmed by real-time qPCR. The level of every gene in the control was set at 1.0. Error bars the standard deviation of three replicates. The values with different lowercase letters are significantly different at the *P* < 0.05 level. **(A)** The relative expression of miR395a/b with the predicted targets CCG007855.1 and CCG033595.2; **(B)** The relative expression of miR475a-3p with the predicted targets CCG020392.1; **(C)** The relative expression of miR6421 with the predicted targets CCG011696.1; **(D)** The relative expression of miR-n87 with the predicted targets CCG033430.1 and CCG0327391.1; **(E)** The relative expression of miR408-5p with the predicted targets CCG004054.1, CCG009428.1 and CCG032972.1; **(F)** The relative expression of miR408-3p with the predicted targets CCG027148.1 and CCG033426.1; **(G)** The relative expression of miR482a.1 with the predicted targets CCG010532.1; **(H)** The relative expression of miR-n10 with the predicted targets CCG020696.1; **(I)** The relative expression of miR-n49 with the predicted targets CCG008392.2; **(J)** The relative expression of miR6462a-3p with the predicted targets CCG012018.1 and CCG014959.1.

The expression levels of predicted targets were measured by real-time qPCR to study whether the predicted target genes were actually regulated by corresponding miRNAs (Figure 7). Sixteenth predicted target genes for 10 miRNAs, which have a low borderline score with 2.0–3.0 (Supplementary Data 6), were selected randomly for the real-time qPCR. CCG008392.2, Cytochrome b/b6, a predicted target of miR-n49 were up-regulated with ABA for 1 day and down-regulated for 4 days. CCG004054.1, Carotenoid oxygenase; CCG009428.1, Cytochrome b5 (Cb5); CCG032972.1, Spermine synthase, were all predicted targets of peu-miR408-5p, were first down-regulated with ABA for 1 day and then up-regulated for 4 days. CCG027148.1 and CCG033426.1, two targets predicted of peu-miR408-3p, which was the complementary sequence of peu-miR408-5p, also both were also first down-regulated with ABA for 1 day and then up-regulated for 4 days. And also, one of predicted targets of peu-miR482a.1, CCG010532.1, Gos1; one of predicted targets of miR-n10, CCG020696.1, Cation/H^+^ exchangers (CAXs), and one of predicted targets of peu-miR475a-5p, CCG020392.1, vesicle associated membrane protein 7 (VAMP7), were all down-regulated with ABA for 1 day and up-regulated for 4 days. Two predicted targets of peu-miR6462a-3p, CCG012018.1 and CCG014959.1, ROPs (rho GTPases from plants), and two predicted targets of miR-n87, CCG033430.1, PP2C, CCG032739.1, drought-induced protein19 (Di19), were induced by ABA. Conversely, two predicted targets of peu-miR395, CCG007855.1, Sulfate adenylyltransferase, and CCG033595.2, sulfate transporter, and predicted target of peu-miR6421, CCG011696.1, *Sec22*, were both inhibited by ABA. The expression profiles of miRNAs and their target genes were complementary.

## Discussion

### High-throughput sequencing of *P. euphratica*

Compared with other deep sequencing studies for *P. euphratica* before, the *P. euphratica* genome was utilized as the reference genome instead of *P. trichocarpa* genome in this study. Finding additional new *P. euphratica*-specific miRNAs was desirable here. Consistently, 94.7% (89/94) of novel miRNAs were not identified in other *P. euphratica* studies before, where *P. trichocarpa* genome was used as reference genome. Only 5 out of 94 novel miRNAs (miRNA-n5, miRNA-n7, miRNA-n33, miRNA-n34, and miRNA-n82) had been found (Li et al., 2013; Si et al., 2014; Table 4). And more novel *P. euphratica*-specific miRNAs, which do not exist in the *P. trichocarpa* genome, were successfully discovered. All of the novel miRNAs discovered had not been reported in other previously studies in *P. trichocarpa* or registered in miRBase (Puzey et al., 2012; Shuai et al., 2013). Our results indicate that it is a more useful approach to discover *P. euphratica*-specific miRNAs based on the *P. euphratica* genome used here.

**Table 4:** **Novel miRNAs found in other studies**.

**miRNA**	**Name in others**	**Found by others**	**Squences (5′–3′)**
miRNA-n5	peu-sM54L	A	GCAUGAGGGGAGUCACGCAGG
miRNA-n7	peu-sM36L	A	GCGGCAGCAUCAAGAUUCACA
	peu-sM44R	A	
miRNA-n34	peu-sM132L	A	GGUCAUGCUCUGACAGCCUCACU
miRNA-n33	peu-sM111R	A	UUUCGGGAAGUGAAAUUUGGA
	peu-MIR343	B	
miRNA-n79	peu-sM64L	A	UUUGGAAGGAAGAUUUGAAGU
	peu-sM70L	A	
	peu-MIR350	B	

A: Si et al. (2014); B: Li et al. (2013).

Several conserved ABA-responsive miRNAs in this study, also significant differentially expressed under drought or salt in previous studies in *P. euphratica* (Li et al., 2013; Si et al., 2014). For example, the expression level of two miRNAs, peu-miR1446 and peu-miR319a, significant changed under salt treatment; four miRNAs, peu-miR399c, peu-miR403-3p, peu-miR472a, and peu-miR6421 were all expression significant changed to respond to drought, other nine miRNAs, including peu-miR156a, peu-miR160a-3p, peu-miR164a, peu-miR168a-3p, peu-miR171c-3p/d-3p, peu-miR395, peu-miR408, peu-miR472b, and peu-miR475, were all differentially expressed under both salt and drought. These observations revealed that some conserved miRNAs respond to multiple forms of stress, and that complex miRNA effects are involved in resistance to abiotic stress in *P. euphratica*.

miRNAs have been shown to play an important role in abiotic stress responses in plants (Ferdous et al., 2015), and ABA is a core signal for abiotic stress responses (Lee and Luan, 2012). The analysis regarding the mechanism between ABA and miRNA at a genomics-level had not been reported previously. In total, 79 conserved miRNAs and 31 novel miRNAs were differentially expressed in response to ABA. Many conserved plant miRNAs regulatory mechanisms have been previously reported, which were also discovered here. For example, a negative regulator in responses abiotic stress, no apical meristem (NAM), was targeted by miR164, which was upregulated by ABA (Souer et al., 1996; Wang et al., 2009; Puranik et al., 2012; Fang et al., 2014). In addition, miR395 targeted two different groups of genes, ATP sulfurylases and SULTR2;1, which are both involved in sulfate translocation and assimilation (Jones-Rhoades and Bartel, 2004; Allen et al., 2005). Targets of all discovered miRNAs and differentially expressed miRNAs were both predicted and subjected to a functional analysis. Predicted target genes of differentially expressed miRNA were more focus on response to ABA addition, with the help of further study that may provide us insight into the molecular mechanism underlying ABA in *P. euphratica*.

### Photosynthesis and stomata movement

Photosynthesis was one of the most sensitive physiological processes responsive to ABA. ABA significantly influenced the transcriptional abundance of genes involved in photosynthesis (Yamburenko et al., 2013; Mou et al., 2015). Protection of the photosynthesis apparatus is very important for stress tolerance (Xiao et al., 2015). Plants can survive severe drought by maintaining the photosynthesis ability to avoid carbon-starvation (McDowell et al., 2008). Similar findings were recorded indicating that *P. euphratica* maintained high photosynthetic rates under moderate drought stress levels (Chen et al., 2006; Tang et al., 2013). In the present study, we found that the photosynthetic rate of *P. euphratica* first decreased and then increased. At 4 days of treatment, the photosynthesis rate of the treated seedlings was higher than that of the control. The internal CO_2_ concentration, however, always decreased suggesting that after 1 day of treatment, stomatal limiting was the most important factor for photosynthesis and transpiration rate, corroborating previous studies (Chaves et al., 2009). After 4 days of treatment, the increase of photosynthesis consumed more CO_2_, and then loaded internal CO_2_ concentration continued to decline. Even when the stomata were still mildly closed, with the transpiration rate lower than of control, the photosynthetic rates still remained at high levels. ABA is a phytohormone, and exogenously supplied ABA can be considered simulating the stress that caused by ABA increasing, suggesting that *P. euphratica* could maintain a high photosynthetic ability to reserve the energy and materials necessary for stress adaptation.

Although variance is correctly used it might be confusing as it is a term used in statistics, variation or changes would be more adequate. ROP2 inhibited ABA-induced stomatal closure (Lemichez et al., 2001; Hwang et al., 2011; Miyawaki and Yang, 2014). Two ROPs were the predicted target of peu-miR6462a-3p. The expression of peu-miR6462a-3p decreased in response to ABA, while ROPs were up- regulated (Figure 7J), which would lead to ABA-induced stomatal closure. That agreed with the phenomenon in this study that stomatal conductance recovered after 4 days of treatment. CAXs involved in calcium (Ca^2+^) transport and homeostasis (Conn et al., 2011; Manohar et al., 2011; Punshon et al., 2012). CAX was a predicted target of miR-n10. After ABA treatment 4 days, miR-n10 was downregulated by ABA, while CAX was upregulated (Figure 7H). In addition, due to CAX genes playing a critical role in sequestering Ca^2+^ into vacuole (Barkla et al., 2008), the increasing of CAX resulted in stomatal opening, which is agreed with the stomatal conductance in this study. And it can been speculated that the regulation mechanism between miRNA and stomata movement may exist, which need further exploration.

### miRNA take part in ABA synthesis and metabolism

ABA is one of the final products of carotenoids (Schwartz et al., 1997; Wang et al., 2013). Carotenoid oxygenase was one of the peu-miR408-5p predicted targets. As peu-miR408-5p was upregulated by ABA after being treated for 1 day, while carotenoid oxygenase was downregulated (Figure 7E), which would influenced the synthesis of ABA. The group A PP2C interacted with SnRK2. And without ABA, SnRK2 is inhibited by PP2C; with ABA, PP2C binds to the receptor to release the inhibition SnRK2 (Umezawa et al., 2009; Ng et al., 2011; Soon et al., 2012). MiRNA-n87 was downregulated by ABA. PP2C was a predicted gene of miRNA-n87, while PP2C was upregulated (Figure 7D). PP2C interacted with SnRK2, and ABA inhibited the reaction—the feedback regulation for ABA and PP2C. It can be speculated that miRNAs may be involved in balance the ABA level and metabolism, and more studies will be needed in the future to address this issue.

### miRNA involved in the crosstalk between ABA and other phytohormone

Overexpression of Cb5s confers lower ethylene sensitivity (Chang et al., 2014). ABA negatively regulated ethylene production (Dong et al., 2015). The two stress-induced hormones ABA and ethylene interacted each other in stomata movement (Wilkinson and Davies, 2010). Cb5 was predicted target of peu-miR408-5p, which increased significantly after ABA treatment 1 day, and Cb5 was increased in expression (Figure 7E). As Cb5 may affected ethylene signaling, suggesting that miRNAs may facilitate another way for ABA to inhibit ethylene signaling. Auxin response factor (ARF), the repressor of indole-3-acetic acid (IAA), decreased in expression in response to ABA. Exogenous IAA increased sensitivity to ABA in Arabidopsis. Furthermore, overexpression of miR160 reduced sensitivity to ABA (Liu et al., 2007). MiR160 appears to promote auxin activity by suppressing the levels of the ARF (Nizampatnam et al., 2015). ARF was the target of miR160 in plant (Liu et al., 2007; Turner et al., 2013; Damodharan et al., 2016; Tian et al., 2016). Therefore, miR160 targeted ARF to decrease ARF-mediated IAA-induced ABA hypersensitivity. In this study, six targets of peu-160b-5p involving ARF were downregulated by ABA at 4th day, corroborating the findings of previous studies. In general, miRNA may involves a very complex set of the crosstalk between ABA and other phytohormone.

### miRNA regulated SNARE interactions in vesicular transport

In this study, peu-miR6421 expression was first increase and then decreased to response to ABA. *Sec22*, a synaptobrevin, was one of the predicted target genes that participated in soluble *N*-ethylmaleimide sensitive factor attachment protein receptor (SNARE) interactions in the vesicular transport pathway (Supplementary Figure 2). In addition, Gos1 and VAMP7 were the predicted targeted of miR482a.1 and miR475a-5p, respectively. They were both upregulated by ABA (Figure 7G) and also involved in the vesicular transport pathway. Sec22 played an essential role in early secretory traffic between the ER and the Golgi (El-Kasmi et al., 2011). In Arabidopsis, VAMP721 and VAMP722 protein levels were downregulated by ABA, leading to slow down plant growth; (Yi et al., 2013). A reasonable speculation was that this inhibited expression by miRNA, so further study was needed.

### miRNA involved in stress-related genes regulation

Di19s were upregulated by the supply of ABA (Li et al., 2010b), and it induced sensitivity to ABA and tolerance to stress in Arabidopsis and rice (Li et al., 2010a; Qin et al., 2014; Wang et al., 2014). Di19 was one predicted targets of miR-n87, which was reduced by ABA. While Di19 was induced (Figure 7D) to improve stress resistance, an assumption was consistent with that of pervious reports. Spermine was a part of polyamines. Free spermine accumulation was showed as a particular metabolic feature of being under long-term salt stress (Maiale et al., 2004). Meanwhile polyamines had been shown to be an important part of plant responses to improve stress resistance (Shi and Chan, 2014; Tiburcio et al., 2014). Spermine synthase was a predicted target of miRNA408-5p, which was significant upregulated by ABA at 1 day, while spermine synthase was inhibited (Figure 7E), which resulted in free spermine accumulation to affect stress resistance. But more evidence was needed to support the interaction between miRNAs and the predicted targets.

## Conclusions

We constructed nine sRNA libraries based on *P. euphratica* leaves for high-throughput sequencing. In total, 151 unique mature sequences belonging to 75 conserved miRNA families were identified. Meanwhile, 56 novel miRNAs of 94 sequences were discovered. Among them, the expression levels of 31 conserved miRNAs belonging to 22 families were significantly different. Confirmed by real-time qPCR, the expression profiles of miRNAs and their predicted target genes were complementary. Based on function analysis, we suggest may play critical roles in maintaining a high photosynthetic ability to facilitate adaptation to stress. And involved several pathways and cellular processes that help this plant to cope with stresses. How individual genes participate in stomatal closure, photosynthesis and other processes involves a very complex set of mechanisms. Our results provide a foundation for further analyses of plant miRNA responses to ABA, and provide new insight into the mechanism underlying the role of ABA in the abiotic stress response and other biological processes, in *P. euphratica*.

## Author contributions

HD, XX, and WY designed experiments; HD, XL, and CL carried out experiments; HD and YA analyzed experimental results. HD and XX wrote the manuscript.

### Conflict of interest statement

The authors declare that the research was conducted in the absence of any commercial or financial relationships that could be construed as a potential conflict of interest.
