# Supplementary material for: Genome-Wide Analysis of MicroRNA Responses to the Phytohormone Abscisic Acid in Populus euphratica
Source: Front Plant Sci. 2016 Aug 17;7:1184. doi: 10.3389/fpls.2016.01184 (PMC4988358; doi:10.3389/fpls.2016.01184)
Supplement: Supplementary file 8 [file Image1.PDF]

Supplementary Figure 1 The predicted stem-loop structures of all the 94 novel miRNAs precursors

(The mature miRNA and miRNA* sequences are colored in red and blue, respectively.)

miR-n1 ΔG = -57.80 kcal/mol

```
          10        20          30        40           50          60         70
-  U      A      A    .-CUUUC|   U   UU     GA--     AG-     .-GACU      -     A
 GG AGUUCG UCGUGG AUUU       UUUC GUA  UCUG    AUAUG   UGUGC      UGUUAU AAUGG \
 CC UCAGGC AGCGCC UAAA       AAAG CAU  AGAU    UAUAU   GCACG      AUAAUA UUAUC U
A  -      C      C    \ -----^   -   U-    AAAG     AAU     \ ----      G     C
   240       230                     180       170       160         80
```

```
      90       100
        AU GG     UCU
--CAGAAA  G  UCUGUCA  U
  GUUUUU  U  AGAUAGU  A
\       GG AG     UCU
        120       110
```

```
 130       140
  ACUUCG         UA
        UCAGAUAUU  U
        GGUCUAUGA  U
  A-----         UC
              150
```

```
190              200
 UAUUA-------   C    A
             GAG UAUG \
             UUC AUAC U
 CUUAUAAUUCAA   -    U
  220       210
```

miR-n2 ΔG = -38.40 kcal/mol

```
        10        20        30        40
|      U      AC      A   AGG      GAA  AA
GGGAAUG AGCCUG  UCGAGA AAC   UAAACU   GG  \
CCCUUAC UCGGAC  GGCUCU UUG   AUUUGA   CC  U
^      U      CA      C   AGA      GG-  GG
   80        70        60        50
```

miR-n3 ΔG = -33.70 kcal/mol

```
          10        20        30
--|  U   C     U      C  A     U
  CGC CGC AGCGU GCACCA CA AACUU C
  GCG GCG UCGCA CGUGGU GU UUGAA A
CA^  U   -     C      A  G     G
  60         50        40
```

miR-n4 ΔG = -44.00 kcal/mol

```
        10        20        30        40
U|                    C              A
 UAGUCUUAAUAAUUGUGUGAUU UCAUAUAUAUAUAU U
 AUCAGAAUUAUUAACACACUAA GGUAUAUAUAUAUA A
A^                    C              U
.        70        60        50
```

miR-n5 ΔG = -46.60 kcal/mol

```
        10        20        30        40
U|   C         GA      UC   GA  C       AG
 CUGC UGGCUCCCU  AUGCCA  UAG  AG UUGUCAA  A
 GACG ACUGAGGGG  UACGGU  AUC  UC AACGGUU  G
G^   C         AG      CA   UC  C       GU
     80        70        60        50
```

miR-n6 ΔG = -52.20 kcal/mol

```
          10         20         30         40          50         60
--                      UC  AC         CCU     -     AGAA    U|  UU   C
  UGUUUCUUGCUCAAAUGAGUAU  CA  AACAGUAG   UUUG AACA    CAUA GUU  UGC \
  AUAAAGAACGAGUUUACUCGUA  GU  UUGUCAUC   AGAC UUGU    GUAU UAG  ACG U
UU                      GA  CU         UAU     G     A---    -^  U-   U
       120        110        100        90         80              70
```

miR-n7 ΔG = -46.80 kcal/mol

```
          10         20           30           40         50
--      G           A  .-AACUUUAAGGCAU   U--|       UAC
  GCGGCA CAUCAAGAUUC CA               GAGU   GGGGGUGG   \
  CGUCGU GUAGUUCUAAG GU               CUCA   CCUCCACU   A
UA      A           G  \ -------------   UUU^       GGC
       100        90                          60

                                          70
                                         ---     G
                                            GAAAG U
                                            CUUUC U
                                         AGU     C
                                             80
```

miR-n8 ΔG = -78.90 kcal/mol

```
            10            20        30        40        50        60          70        80         90        100
--     -    -          ----   U        UC  G     CUUA   CU          .-ACCUACA|  ACAAACC  UG  -   UC   U  GC-    CAUA
  CAGGC GGUC UCCUUGGCUA    ACA AGGCUCUU   CU UCAUG    GCC  UCUCUUCUUC         GCU       CC  AU CAU  UAC GG   GUCG    U
  GUCCG UCAG AGGAACCGAU    UGU UCUGAGAA   GA AGUAC    CGG  AGAGAAGGAG         CGA       GG  UA GUG  AUG UC   UAGC    A
UC     U    U          AGUU   -        UA  -     AUUC   --          \ -------^  CC-----  GU  C   GU   -  AUA    AACU
   210       200       190        180        170         160                             130       120        110

                                                                    140
                                                                      AA   A
                                                                        UCA A
                                                                        AGU C
                                                                      --   U
                                                                       150
```

miR-n9 ΔG = -41.40 kcal/mol

```
          10          20        30
--                    |       UUU     GU
  UCAGGGCACUGCAAUUCU--AAAUGUCU   GGCAU  G
  AGUUCCGUGACGUUAAGA  UUUGCAGA   CCGUA  G
CU                  \ ^       ---     GC
       90        80     50           40

                           60
                      GAAAUUUAC   UC
                               AUA  \
                               UAU  A
                      CUGAU----   UU
                                 70
```

miR-n10 ΔG = -27.50 kcal/mol

```
          10        20        30
--|   GC         A    U      UUGUUC
  GAAU  UGACCGAAU UGGA GAAAAG      U
  CUUG  ACUGGCUUA ACCU CUUUUC      C
ACˆ   UA         C    C      CUUUGU
.        60        50        40
```

miR-n11 ΔG = -23.40 kcal/mol

```
          10        20               30
--       CCA         A-  -----    -| C   AUG
  UCAAUGG   UUGUAAGAG  GA     AGGA UC AUG   C
  AGUUACC   GACAUUCUC  CU     UCCU AG UAC   A
UU       UGA         AC  UAUAG    Cˆ U   AAA
80        70        60        50        40
```

miR-n12 ΔG = -30.40 kcal/mol

```
          10         20        30        40
--| A   UU   -   CU        A    AAGA    CUC
  GG AUG  GGC UGG  CGAAGCUU AGCA    GUUUC   \
  CC UAC  UCG ACC  GCUUCGGA UUGU    CAAAG   U
CCˆ C   U-   G   AG        A    CAA-    AAC
   80          70        60          50
```

miR-n13 ΔG = -30.90 kcal/mol

```
         10        20                  30
-       UG     A   C      ---------|   C  UC
 GUUCCUC  AGCAC UCA UGGGGA         UCUU CA  G
 CAAGGGG  UUGUG AGU AUCCUU         AGAA GU  A
U       GU     A   C      ACUAUUAUGˆ   A  CC
.        70        60        50        40
```

miR-n14 ΔG = -62.20 kcal/mol

```
          10        20        30          40        50
AA--     A    C          CACU        .-AUGCACACAGA   GG GA
    GAUGG GAAG AGGGCACGUG    ACUAACUC             GAG  A  C
    CUACC CUUC UUCCGUGUAC    UGAUUGAG             UUC  U  G
AGUA     C    U          UUAU        \ -----------   UU AC
  150       140       130       120                  60
```

```
                                           70         80        90
                                                -     UUA|  C  UUA
                                       GCUGGAGCU ACGAG   CGA UC    C
                                       UGACCUCGA UGUUU   GUU AG    C
                                                U     UA-^  -  CAU
                                            110        100
```

miR-n15 ΔG = -41.10 kcal/mol

```
            10        20          30
----               A       UUCG--|GG   CACC
    UGAUGACUGAUCUUG GCAUGCU      G  AGG    \
    GCUACUGGCUAGAAC CGUACGA      C  UCC    A
ACGA               C       UGAUAA^UU   CUAU
80        70        60        50        40
```

miR-n16 ΔG = -94.90 kcal/mol

```
            10                  20        30        40          50
--                   ----------|      G  CC            .-CCUUGUUUCU      A
  GUGGGCUU--GUGAUCCAA          GUGGAA CA  GAGGUGACGGGA            AUCCAG A
  CACCCGAA  UAUUAGGUU          UAUUUU GU  CUUUAUUGUUUU            UGGGUC G
UC        \          UUUAUUUAUU^      A  UU            \ ----------      A
     280           230       220       210       200                   60
```

```
               70        80        90          100        110        120
UU-------------        UAUUCUCU     AC---  A     A-   UA        U    C  GUUUCACA
               GUGGCGGC        GGCCU     CC UUCCA  GCC  AAGGGUG GAGG UG         \
               CACCGUCG        CCGGG     GG AAGGU  CGG  UUCUCGC CUCC AC         C
UUCAAUACUUGUUGU        --------     CAAUA  -     AA   --        C    A  AGCUACUA
     190       180               170        160         150       140       130
```

```
          240               250
            A------   --    G     A
                   CAG  GUU UCCGU G
                   GUC  UAA AGGUA U
            GAGUAAG   GA    G     U
                 270       260
```

miR-n17 ΔG = -39.50 kcal/mol

```
          10        20         30            40
-  -                  -  AAAUUC   UG    ----|  AUG
 GG UAUCUACAGUAGCCAAGC AC       GCA  GAA    GGA   U
 CC AUAGAUGUUAUCGGUUCG UG       CGU  UUU    UCU   G
C  A                  U  GACGUC   GU    UGUC^  CUC
  90        80        70        60        50
```

miR-n18 ΔG = -33.20 kcal/mol

```
        10        20          30
   U  UC           U        .-U|  A
GGA UG  GUCUGGUUCGA GUCAUUCA    GAG \
CCU AC  CGGACCAGGCU UAGUAAGU    CUC A
   U  UU           U        \ -^  G
  80        70        60

                              40
                              AA---  AU
                                   AC  A
                                   UG  A
                              UAUAA  CA
                                  50
```

miR-n19 ΔG = -39.70 kcal/mol

```
          10        20          30
UC--              A        .-CCU|  U    UG
    AUUGAGUGCAGCGU GAUGAAAU     ACA UUUG  C
    UAACUUACGUCGCG CUACUUUA     UGU AAAU  U
GUAC              A        \ ---^  C    UA
  100        90        80       50        40

                                        60
                             UA--   A-    U
                                 CCA  CCCU U
                                 GGU  GGGG G
                             CUAC   AC    U
                                   70
```

miR-n20 ΔG = -42.30 kcal/mol

```
             10        20        30          40        50
-----   A          A     U  CC     .-UAAGUCUU|      U   UAU
     CAC GCUUUCUUGA CUUCC UG  GUGCU          GUGUGUG GUG   \
     GUG CGAAAGAACU GAAGG AC  CACGA          UAUAUAU CAC   A
AGAGG   C          C     U  --     \ --------^      -   UAG
     120       110       100                      60

                                       70        80
                                     CUGUA      UUC
                                          UAGUUA   U
                                          AUCGAU   U
                                     UCG--      UUG
                                          90
```

miR-n21 ΔG = -43.80 kcal/mol

```
          10        20        30
--     C            C   U      C    ----| CC
  GGAGG AUCCAAAGGGAU GCA UGAUCC GAGU    GU  \
  CCUCC UAGGUUUCCCUA CGU ACUAGG UUUA    CG  C
UU     U            U   -      U    GUAC^ GU
 80        70        60         50        40
```

miR-n22 ΔG = -61.00 kcal/mol

```
          10        20        30        40         50        60        70            80         90         100
--        U   U        GCCCGU   AAGC          AU   -     GCU   AAGA      --| UGU   --         -    AA       AUU
  GUUCCUCC AGC UCUUCAGU      GGA    UAAUGCUACA  UAU CUUGA   GGU    UUGGCA  GG   CAG  AUUUGGAUG UGUU  AGGUAAU   \
  CAAGGAGG UUG GGAAGUCG      CCU    AUUAUGGUGU  AUA GAACU   UUA    GAUUGU  UC   GUU  UGAACUUAC GCGG  UUUAUUA   A
CU        U   -        UUUAGU   C---          --   U     AAU   GGA-      GA^ UU-   GA         C    CA       GUU
     200        190       180          170         160       150        140        130        120        110
```

miR-n23 ΔG = -33.10 kcal/mol

```
         10        20                 30
-       UG         AC     ---------|   C  UC
 GUUCCUC  AGCACUUCA  GGGGA         UCUU CA  G
 CAAGGGG  UUGUGAAGU  UCCUU         AGAA GU  A
U       GU         CA     ACUAUUAUG^   A  CC
.        70        60        50        40
```

miR-n24 ΔG = -54.90 kcal/mol

```
        10        20         30         40        50
CU     UC          U       -    -| GGUAA       UCU       AA
  CCCCC  AAGGGCUUCC GUUUGCC CGAU CU     UGAUGGU   GUGUUUU  \
  GGGGG  UUCUCGGGGG CGGACGG GCUA GA     ACUAUCA   UACAAAA  G
G-     UU          U       A    U^ -----       UG-       CA
.        100        90        80             70         60
```

miR-n25 ΔG = -25.70 kcal/mol

```
          10         20        30        40
--      UU         A   -|C  GCCACCCC        GA
  GGAAUG  GUUUGGUUC AGG C UG        AUGUUUUG  \
  CCUUAC  CGGACCAGG UCC G AC        UAUAAAAU  A
CU      UU         C   U^U  --------        UU
 80        70        60                50
```

miR-n26 ΔG = -28.60 kcal/mol

```
           10         20        30
---       U      A          CACAAC--|  UCA
   AGUUUGU CGUGGA CUGAUGCCAU        CGU   \
   UCAAACA GCACUU GAUUGCGGUA        GCA   U
CGC       C      A          ACCUACCA^  CUU
.        70        60        50        40
```

miR-n27 ΔG = -89.90 kcal/mol

```
                10         20         30         40         50         60            70         80         90        100        110        120
-----         UG         ACU          C          CA   AUA    C  U  -        A--|     G        U   A       A      CC         A        ACAAA
     UCGAGAUC  UUCAAUAGU   AGUGGUGUUAU AUCGGAGCUU  AU   CCU UA GA UCUUUUU   UUUUU UUUUUAU UUU UUUUUU CUUUU  UCUCUUCU UCUCUUC      U
     AGUUCUAG  AAGUUGUCA   UCACCAUAGUA UAGUCUCGAA  UA   GGA AU CU AGAAAAA   AAAAA GAAGAUA AAA AAGAGA GAGAA  AGAGAAGA AGAGGAG      U
AGAAC         CA         CGU          A          AC   CAC    A  -  C        GAA^     G        C   -       -      U-         G        GAACA
      240        230        220        210        200        190         180        170        160          150         140        130
```

miR-n28 ΔG = -36.40 kcal/mol

```
              10         20         30          40         50
---         C        C          ACU   --|     AGUGUCUUUGUU
   UAUUGGC UGGUUCA UCAGAUCACG   UCA  AAGCAA             \
   AUAACCG GCCGAGU AGUUUAGUGU   AGU  UUUGUU             C
UCU         U        U          GCU   AU^     CUUCUUCUUCU
     100        90         80         70         60
```

miR-n29 ΔG = -51.30 kcal/mol

```
                10         20             30          40         50         60
    --      U               UU---    UU--     .-GUUUUUUA|      A  AAA    AAGU
      UGCAUU GCACCUGCAUCUUA     GCUU     GUUUC           CUCCACA AC   AUC     U
      ACGUGA CGUGGACGUGGAGU     CGAA     CAAAG           GGGGUGU UG   UAG     C
    GG      U               UGAUU    CAAU     \ --------^      A  ACA    CUGU
     .         140        130        120                        80         70


                                                                      90
                                                                CA-    A     A
                                                                   AUU GAUAU \
                                                                   UAG UUGUG G
                                                                GUA    A     A
                                                              110        100
```

miR-n30 ΔG = -35.30 kcal/mol

```
          10        20           30
U--    CU             C      CC---    -| G
   AAUG  GUCUGGUUCGAGA CAUUCA     UGAA GC C
   UUAC  CGGACCAGGCUCU GUGAGU     ACUU CG A
CCC    UU             A      UUUCU    A^ C
.        70        60        50        40
```

miR-n31 ΔG = -42.00 kcal/mol

```
          10         20        30        40
U-      -     -|        A        UAUUU     A
  UGACAG AAGAG AGUGAGCAC CAGAGGCA     GUAUA A
  ACUGUC UUCUC UUACUCGUG GUUUUCGU     CAUAU A
UC      U     U^        C        UAC--     U
    80        70        60        50
```

miR-n32 ΔG = -36.90 kcal/mol

```
         10        20         30
U-     UU         A   -|   GC     CAU
  GAAUG  GUCUGGUUC AGG CCUG  CACCA   \
  CUUAC  CGGACCAGG UCC GGAU  GUGGU   C
CC     UU         C   U^   AA     UUU
  70        60        50        40
```

miR-n33 ΔG = -35.80 kcal/mol

```
           10        20        30              40             50
--    -          A   A       C    -----   -       -----|    A U
  CAAA UUCACUUCCC AAA AUUUCAA CAUC     GGA UUUAUGU     GAUUA C A
  GUUU AAGUGAAGGG UUU UAAAGUU GUAG     UCU AGGUACG     CUAGU G G
AG    A          C   C       C    GUUAC   C       UUCAA^    - U
 110       100        90        80        70        60
```

miR-n34 ΔG = -31.00 kcal/mol

```
              10          20         30            40
    --   A    C --           U  UU      AGAA---|  AU
      UGA GCUG C  AGCAUGAUCUA CU  GGUUAG       GU  A
      ACU CGAC G  UCGUACUGGAU GA  CCAAUC       CG  G
    UC   C    A UC           C  UC      AAGAAAG^  AA
            80         70         60         50
```

miR-n35 ΔG = -58.90 kcal/mol

```
              10         20         30         40         50          60         70         80         90         100
    --          G   U      U       AUCAAC    CUC       C    .-AAA|  CCAC     UUAU          A   A     -    GUAA
      UGAGAUCUUUU AAU AUAUCA UAAUGUC      GAAG   CGAUGUG CUUU     UGA    AUUUG    GUUUUUUUUU UCU UUUUC CUU    U
      AUUUUAGAAAG UUG UAUAGU AUUACAG      CUUU   GCUACAC GAGG     ACU    UGAGC    UAGAAAAAGA AGA GAGAG GAA    U
    CA          G   U      U       CAAUAA    AAA       U    \ ---^  ACAC     UU--          A   A     A    AUGU
     230        220        210        200        190        180           140         130        120        110
```

```
       150
     AAACU--       G
            UUAAUAAU \
            AGUUAUUA A
     CUCUUAU       C
         170
```

miR-n36 ΔG = -32.00 kcal/mol

```
             10         20         30
    -|                    C    ACCUAACAAUC
     CAAGUAAUUCUACCAUCAAUUC UAAA           \
     GUUCAUUAAGAUGGUAGUUAAG AUUU           A
    G^                    A    AAAACAAACUU
           70         60         50         40
```

miR-n37 ΔG = -48.30 kcal/mol

```
          10        20        30
--|                           CUCUCUCUC
  AUAAAGUGGGCAGUAGAGAGUAAGCUCU         U
  UAUUUCACCCGUCAUCUCUCAUUCGAGA         C
UC^                           UAUAUAUAU
       70        60        50        40
```

miR-n38 ΔG = -47.30 kcal/mol

```
          10        20
--|               C         GAA
  CCAAUAUCUGGAAGGG UGGGUGCCA   \
  GGUUAUAGACCUUCCC ACCCACGGU   A
GU^               A         GUU
60        50        40
```

miR-n39 ΔG = -79.20 kcal/mol

```
           10        20        30        40           50        60        70        80        90
A---|    UU      C   CU      U      AA    U   ---    G  G   AC        U   G   U     AA       GAAU
    GAGCU  CUUCAG CCA  CAUGGA AGGAGA  GGGG UGA   AUUA CU CCG  UCAUUCAU CAA CAC AGUAG  AAAAGGG    \
    CUCGA  GAAGUC GGU  GUGUCU UUUUCU  CCCC ACU   UAAU GA GGC  AGUAAGUG GUU GUG UCAUC  UUUUCUU    G
CUUC^    GG      A   UC      -      --    U   UUU    A  G   GU        U   A   -     G-       AUAG
  180       170       160        150         140       130       120       110         100
```

miR-n40 ΔG = -39.90 kcal/mol

```
           10        20        30         40
---    A                   AGAAAAG-|   AAA    UC
   AGUC AGAGAUACAUGAACAGCCG        UAGA   GGUU  A
   UCAG UCUCUAUGUACUUGUCGGU        AUCU   UUAG  A
UUC    C                   AAAUUAUA^   AG-    UA
  90        80        70        60         50
```

miR-n41 ΔG = -51.10 kcal/mol

```
         10        20         30
-                            -|  A   A
 AUUUGCUUGUAUUUAACUCCCGCCCCGU UGU UUG C
 UAAACGAACAUAAAUUGAGGGUGGGGCA GCA AAC A
A                            U^  -   C
    70        60        50         40
```

miR-n42 ΔG = -64.80 kcal/mol

```
           10        20         30        40          50          60        70        80
--   -   U     U      UGA-          U   U     -  UA-  U       A--|   UA     AAAUAG   GA     GUA
  UGA AGA AAGAG CUUGUU    UAGCCAAGGA GAC UGCCU GC   CA ACAAGAG   UCAU  UGCAU      AAC  AAUAU   \
  ACU UCU UUCUC GGACAG    AUCGGUUCCU CUG ACGGA CG   GU UGUUCUC   AGUG  ACGUA      UUG  UUAUG   C
GU   C   C     -      UCGA          -   -     C  UUG  U       AAA^   --     A-----   A-     GUC
170       160        150       140         130       120       110              100         90
```

miR-n43 ΔG = -42.60 kcal/mol

```
         10        20          30
-     UU      CU   G      .-UU|      GAG
 GAAUG  GUCUGG  CGA GACUCU    CUUGAU   \
 CUUAC  CGGACC  GCU CUGGGA    GAACUA   U
C     UU      AG   G      \ --^      AUC
.       100        90              40

                              50        60
                            UUUUCUAUCUUUA       U
                                         GAUCUAA A
                                         CUAGAUU U
                            UUGUGUA------       C
                               80              70
```

miR-n44 ΔG = -83.80 kcal/mol

```
               10         20         30         40           50         60
    U---                    GAUCCAGAAA     AUG     .-AUAAA|      AAG
        GGGGCAAAAGCGCAUAUGAGA          GGGAC   GCAAG       UUUUUA    A
        CCCUGUUUUUGCGUAUACUCU          CCCUG   UGUUC       AAAAGU    A
    CGCU                    ----------     ---     \ -----^      GUA
      260        250        240                     230      70
```

```
                                                                      80         90           100          110          120           130
                                                              G    A    AAAA       A  ---        .-CCCGGA  A    --      ---    CA  A  UA
                                                               AAAU AGCA    CUAAGAG GG   AAACUAA        GG GCCU  UGCCAA   CCAG  GC UC  \
                                                               UUUG UUGU    GAUUUUC CC   UUUGAUU        UC CGGA  ACGGUU   GGUU  UG AG  C
                                                              A    G    AUCC       A  CAA        \ ------  -    CU      CAA    --  -  AC
                                                                     220        210        200           160         150          140
```

```
                                                                                                                    170
                                                                                                          AUG---   GUUU    C
                                                                                                                UUC    GCAG U
                                                                                                                GAG    CGUC A
                                                                                                          AGAUUA   AAU-    U
                                                                                                             190         180
```

miR-n45 ΔG = -34.90 kcal/mol

```
              10         20          30
    --         CA           CUCU-| CU
      UGGAGAAG  GGGCACGUGCAAG     CU  \
      ACCUCUUC  CUCGUGCACGUUC     GA  U
    CA         CC           UUUUC^ AC
         60         50         40
```

miR-n46 ΔG = -41.70 kcal/mol

```
           10        20        30
--|     A  -           U   U       -  U
  UGGAGA GC AGGGCACAUGC GAA CUAUCAG CU G
  ACCUCU CG UCUCGUGUACG UUU GAUAGUC GA A
CG^     -  A           U   -       U  A
   70         60        50         40
```

miR-n47 ΔG = -37.70 kcal/mol

```
         10        20        30        40        50        60
-                       UAGUCUUUUAC|     GUAUUG  U  U      UACAA
 UAUUAUAACCAAGACUAAUGCAU           UCUGUU      AU CA AAAUGA     U
 AUAAUAUUGGUUCUGAUUACGUA           GGACAA      UA GU UUUAUU     A
U                       UU---------^     AUUAUA  C  C      UCUUA
.       110       100                 90        80        70
```

miR-n48 ΔG = -62.60 kcal/mol

```
          10        20          30
--                                A
  AAGUGAGUUGCCAGUGUAACCUUACAU--GUC \
  UUACUCAACGGUCACAUUGGAAUGUA   CAG A
AG                           \    A
    180       170       160
```

```
  40        50        60        70             80        90
U     GGGACCCUA    AAUA    GUU   AAU     -----|  CC   A     U   GU
 GGUUU         UCAA    UUAU   GCA   GCUAU     UAU  AUG ACGGC AGU  \
 CCGAA         AGUU    AGUG   CGU   UGAUA     AUA  UAU UGUCG UCA  A
U     AGACAUUAA    A---    GCU   ---     UAUAU^  UA   C     -   GA
     150       140          130          120       110        100
```

miR-n49 ΔG = -42.30 kcal/mol

```
          10        20          30
--          A          .-UGAUG|  UU UA
  CAUAGAACAC CAACCCCGUC       GU   C  \
  GUAUCUUGUG GUUGGGGCAG       CG   G  U
GA          G          \ -----^  GG AU
    80        70              40


                                50
                         AGGAUUCAA    G
                                  CAUC U
                                  GUAG C
                         ---------    A
                                   60
```

miR-n50 ΔG = -36.70 kcal/mol

```
          10        20          30          40         50         60
-    A-   U              -  A      -     AGAAAAA    AAGAA|    GUAUU
 GGGG  CA AAACGCAUAAGGAU CG AGGGGU GCAAG       UCUG     CAAA     A
 CUCC  GU UUUGCGUAUUUCUA GU UUUUCA CGUUC       AGAC     GUUU     A
G    CG   U              U  G      U     AAA----    -----^    AAGAA
       110       100        90         80                   70
```

miR-n51 ΔG = -47.20 kcal/mol

```
          10        20        30
--|                        CCUUCUCCA
  UCCCUUCUCCAGUGUGUGUGUGUGU         \
  AGGGAAGAGGUCACACACACGCACA         G
GA^                        CGUGCGUGU
70        60        50        40
```

miR-n52 ΔG = -84.40 kcal/mol

```
        10           20          30           40         50         60
AAGU   UG UA--    C-      A   .-AACA       ---    A    -   AG    -      AG
    ACC  A    GCAA  UCAAGU ACA      CCUGGCG   CUAG GCCA GUA  UGUG CCUGGU  C
    UGG  U    CGUU  AGUUCG UGU      GGACUGU   GAUC CGGU CAU  ACAU GGACUA  A
----   GU CAAA    UC      -   \ ----       CAA    A    U   AG    A      CG
          270       260               100        90        80        70
```

```
   110       120         130        140       150       160       170
CCCUGA   U    UAAA|  .-ACACGC    CUGA-          A  A     A    CU UCA
      GCU UAGA    CCC        CUUG     AAACUCAACG GU GGUUU UAGA  C   U
      CGA GUCU    GGG        GAAC     UUUGGGUUGU UA CCAGA GUCU  G   G
AAUC--   C    ----^  \ ------    CCAGA          A  A     C    AG UAC
 250             240       210       200       190       180
```

```
       220
AAAGUA    U   A
      GUUC ACA A
      UAAG UGU C
AGC---    -   U
          230
```

miR-n53 ΔG = -68.60 kcal/mol

```
             10        20        30          40        50        60        70        80        90
---      --        A  C    A  AAA     .-UGAU|             U  CACC        U    AA        UAAGCCCCUAUAC
   UUAGAG  ACACCAGA CU UGAU AU   ACCAC      ACGACAUUAAGGAA GU    AAUAGUGA CGGA  GGGCCAUA             \
   AGUCUC  UGUGGUUU GA GCUA UA   UGGUG      UGUUGUGAUUUCUU CA    UUAUCAUU GUUU  CCCGGUAU             C
UGG      CG        A  A    C  CUG     \ ----^             C  AUAU        U    CA        CUUUAGAAAGUUU
    220       210       200       190       150       140       130       120       110       100
```

```
                                                      160
                                        --------    CUU-     G
                                                UUGA    GUCUC \
                                                AACU    UAGAG U
                                        UUCAUUAC    UUUC     C
                                              180       170
```

miR-n54 ΔG = -40.20 kcal/mol

```
          10        20          30            40
--     C     GC          AA-   U     ----|     A
  GGGCA CUCUU  UUGGCAAGC   UGG GAUUG    UUGCAA \
  CCCGU GAGAG  AACCGUUCG   ACU CUAGU    GACGUU C
GU     U     GA          GUG   -     UUAA^     A
      80        70        60          50
```

miR-n55 ΔG = -43.20 kcal/mol

```
          10        20        30        40        50
--                           C   AUGAU A      UUUC|    U
  UUGUAAUGAUAUAGAUGUGAUUAAAGU UUC     C UUGCUA    GUUAG \
  AACAUUACUAUAUCUACACUAAUUUCA AAG     G AACGAU    CGAUU U
UU                           A   ----- -      UU--^    A
 100        90        80             70           60
```

miR-n56 ΔG = -33.40 kcal/mol

```
           10        20        30
---|          C         A    AACA   UG A
   UCAAUGGAGCC AGAUGUAGA AUGU    UUU  A \
   AGUUACUUCGG UCUACAUCU UACA    AGA  U U
UAC^          U         G    CUCA   GU U
       70        60        50        40
```

miR-n57 ΔG = -36.00 kcal/mol

```
          10        20
--                      -----|   AU
  UUGAAUAUGCUUGAGCUGUGUU     UCAU  \
  AACUUAUACGAACUCGACACAA     AGUG  A
GA                      ACACU^   AC
   60        50        40        30
```

miR-n58 ΔG = -63.20 kcal/mol

```
         10        20        30        40              50        60        70
-   A      A          ACC                 U   ------| GG      AUUGC      CAG
 UGA AAGACG AAGAGUAGAG   GAGCCAAGAAUGACUUG CGG      CA  CUAGCA     UACGUA   A
 ACU UUUUGC UUCUCGUUUU   CUCGGUUCUUGCUGGGC GCC      GU  GAUCGU     AUGUAU   G
U   C      -          AA-                 U   UUAUGA^ AA      -----      UAC
.       130        120        110       100        90             80
```

miR-n59 ΔG = -43.90 kcal/mol

```
           10        20        30        40        50
---|           C            UGUACAAC      GGAA  GGAA  GA
   AGGGGCAAAAAU GCAUAAGAGAGA        GGGUAG    GA    GA  A
   UCCCUGUUUUUA CGUAUUCUCUUU        CCCAUC    CU    CU  A
UGC^           -            UUCUU---      AG--  AUAG  AG
   100         90        80           70          60
```

miR-n60 ΔG = -68.90 kcal/mol

```
          10        20          30          40        50          60
--          AUA               --       .-CAU   CUCCAU    .-A|    AU   GUG
  AAUCAUGCAU   ACUUAAUAUUAUACU  GAAUGGC     GAG      GGAC   UGUCA  UGC    \
  UUAGUACGUA   UGAAUUAUAAUAUGG  UUUACCG     CUC      CCUG   ACAGU  ACG    C
AG          CGC               UU       \ ---   UCUU--    \ -^    GU   AAA
  230       220       210       200         160                     70
```

```
80                90
 AG------    .-UUUU      UU
         CAGA      UCUUUU  U
         GUCU      AGAAAA  A
 ACUACAAA    \ ----      CA
150       140           100
```

```
110       120
UUCACAUU   C   AC
        AGA AGA  \
        UCU UCU  C
CUCUC---   A   CU
          130
```

```
      170
U-   C    AUCUCU
  CCC GAUG      U
  GGG CUAC      C
UU   A    CUCCUC
   190       180
```

miR-n61 ΔG = -64.30 kcal/mol

```
             10        20        30        40          50        60          70
    -----  U             AUUUA   A   U    AUU          .-AG      G       UCAA    -    U
         UG UGAUGAUGAUAA     UUU GGU AUUA   UUUUCAAAUU    AGAAA AAUUAU     GCUA AUCU A
         AC ACUAUUGCUAUU     AAA CUA UAGU   AGAAGUUUAA    UCUUU UUAAUA     CGAU UAGG U
    UAAGU  -             AUUG-   A   U    AAU          \ --      A       ----    A    A
    290        280       270        260       250                90             80
                                                        100       110        120        130
                                                       .-AUAAUUAUC     UA-|        .-AUUAU     AAGU
                                                                  UAGUU   UUUGGAUAA       ACUUA     \
                                                                  AUCAA   AAACCUAUU       UGAAU     U
                                                       \ ---------     UAG^        \ -----     AGAC
                                                                        180                   140
                                                                                              150
                                                                                     ------      AAA
                                                                                           UUUAUC   A
                                                                                           AAAUAG   C
                                                                                     GAACAU      AAA
                                                                                    170        160
                                                         190       200
                                                        .-UUAAAGAA      U
                                                                  GUGUUU G
                                                                  CACAAA U
                                                        \ --------      G
                                                                  210
                                                              220       230
                                                         AUUAU          AG
                                                              UAGUCUGUUA  A
                                                              AUCAGAUAAU  A
                                                         C----          GU
                                                               240
```

miR-n62 ΔG = -50.70 kcal/mol

```
           10        20          30          40        50
---          U   U     -  UACUU-         --      GCAAAGAGAGA|  AAA
   UAGCCAAGGA GAC UGCCC GC      GCAAGAGUU  UCUGCA           AUG   \
   AUCGGUUCCU CUG ACGGG CG      UGUUCUCAA  AGAUGU           UAC   U
CCA          -   -     C  UUAGUU         AG      AAAUUAAGG--^  GUA
120       110         100        90        80        70
```

miR-n63 ΔG = -47.30 kcal/mol

```
          10        20        30
--|                               G  A
  UUAUAAAGCCAUAAGAAGUCCAAUUCUUUAUU UC U
  AAUAUUUCGGUAUUCUUCAGGUUAAGAAAUGA AG U
UU^                               A  A
     70        60        50        40
```

miR-n64 ΔG = -41.10 kcal/mol

```
        10        20        30
A|                          U
 AGAGACUCUUGUAUGUAUGUAUGUAUG A
 UCUCUGAGAACAUACAUACAUACAUAU U
-^                          G
         50        40
```

miR-n65 ΔG = -43.70 kcal/mol

```
          10        20        30         40
--|        C    A           GG   -  AAAUUAA
  UUCUGUCGC GGAA GAUGGUGCCUA  GCU GC       \
  AAGGCAGCG CCUU CUAUCACGGAU  CGA CG       U
UC^        -    -           AA   A  AUAUGUA
 80         70         60        50
```

miR-n66 ΔG = -67.80 kcal/mol

```
        10        20        30        40        50
A|                      A    C              AAAUU  U
 GAAAGAAGACAGAAGGCAGCGUG UAUU GUGUCUUUCUGUGU     UG U
 CUUUCUUCUGUCUUCCGUCGCAC AUAA CACGGAAAGACACA     AC G
-^                      C    C              CACUU  C
    100        90        80        70        60
```

miR-n67 ΔG = -30.20 kcal/mol

```
          10        20            30
--                      CC----|   AUAA
  ACAUAAAUUGCAAGGAACUUAA      CAAG    U
  UGUAUUUAACGUUCCUUGAAUU      GUUC    U
GG                      UACUAU^   GUAA
 70        60        50        40
```

miR-n68 ΔG = -41.60 kcal/mol

```
          10        20         30            40        50
--     UUCU    U    G  UCAA-     GAGUU---  -|           UA  G
  UCUGU    GGCU GUCU AU     GCCCU        GA ACCAUGGCAUCC  GU A
  AGACA    UUGG CGGA UG     UGGGA        CU UGGUACCGUGGG  CA A
CG     CU--    U    G  CGAUA     GGUGAGAU  A^           UA  C
   110         100        90        80        70        60
```

miR-n69 ΔG = -38.30 kcal/mol

```
           10        20        30        40
CUC---|     C               ACUA   AAA    UAUG
      GAUGAC GAUCUUGGGCAUGCU    UUG   GGGA    \
      CUACUG CUAGAACUCGUACGA    AGC   CCCU    G
UACGAA^     A               A---   ---    CCGU
   80        70        60              50
```

miR-n70 ΔG = -42.10 kcal/mol

```
         10         20                       30
-       UG         C     ---------------|     AG
 GUUCCCC  AGCACUUCA UGGGA               CUCUUU  A
 CAAGGGG  UUGUGAAGU ACCCU               GAGAAA  A
U       GU         C     UAUAACUACUAUAAC^     GU
 80        70        60        50        40
```

miR-n71 ΔG = -31.00 kcal/mol

```
           10         20          30        40
ACUU---      ACUA   -              UU       GUA
       UUAAAU    AUC AAUAUG--GAUAUG  AAUUUU   \
       AGUUUG    UAG UUAUAU  UUAUAC  UUAAAA   U
UGUGCAU      GAUG   A      \       UC       AUA
    160       150       140         50
```

```
    60         70         80        90
U----|   UAAAAA-     UUA-     A        C  GU
     UCCA       CAUGA    AUCCA UACAUAAG AU  \
     AGGU       GUACU    UAGGU AUGUGUUU UA  U
AUUUU^   UACAUGG     UGUG     -        -  AU
       130       120       110         100
```

miR-n72 ΔG = -49.70 kcal/mol

```
          10        20        30          40        50        60          70        80
--               C      A   C   .-UUCUUCAAAGAAACAACUAAAUA  G      .-AGCA   AAA  C   UC
  UUUCAGUUUGAUUCAG AUGCCC AAA GAG                         CU AUAUAG      GGG   AC UUC  C
  AAAGUCAAACUAAGUC UACGGG UUU CUC                         GA UAUAUC      CCC   UG AAG  A
GA               A      C   U   \ -----------------------  A      \ ----   G--  U   UU
 190       180       170                                     120              90

                                                                   100       110
                                                                 AAAAAAAAUUUA  C
                                                                            UC A
                                                                            AG A
                                                                 CAA--------  A

                                       130       140
                                     AACAAAAAAA|  AGAC
                                               UGC    \
                                               ACG    G
                                     CUAAAACGG-^  AGAU
                                   160         150
```

miR-n73 ΔG = -127.00 kcal/mol

```
          10        20        30        40        50        60        70        80        90        100       110       120
--|  A        U         A         A                        U                         C                 CUGUGUUG  U    UU     AU
  AUAA CUUGAUUA GUGGAAUAU UAGUAAAGA AAUCAAAGUUGGUUACCACGACAGA AAGUGAUAUUAGGGUAUUAAAUCU UCUUAUUUUAAUUUUAU        UG UGUG  GUUUU  U
  UAUU GAACUAAU CACCUUAUA GUCAUUUCU UUAGUUUCAACCAAUGGUGUUGUCU UUCACUAUAAUUUUAUAAUUUAGA AGAAUAAAAUUAAAAUA        AC AUAU  CAAGA  U
UG^  C        U         C         C                        U                         A                 AAGAGA--  U    UU     AU
  250       240       230       220       210       200       190       180       170       160       150         140       130
```

miR-n74 ΔG = -41.50 kcal/mol

```
           10         20        30        40
--|      U    -         UG     UC      CUUG       CU
  UGAACUC CUC CCUCAAC  CUAC  UAGAG    GCAAUC  U
  ACUUGGG GAG GGAGUUG  GAUG  GUCUU    UGUUAG  G
AG^      -    U         GU     U-      ----       CU
    80         70         60              50
```

miR-n75 ΔG = -73.30 kcal/mol

```
                10        20        30        40        50
-------|                                                  GAUUCUCA
       UUGCAAAUUACAAAACUUACAAUCUCAAUACACAAUCUCAAAAACACAG        C
       AAUGUUUAAUGUUUUGAGUGUUAGAGUUAUGUGUUAGAGUUUUUGUGUC        U
UUGUUCA^                                                  AUCUCUUU
 120       110       100        90        80        70        60
```

miR-n76 ΔG = -40.70 kcal/mol

```
           10         20        30        40
--           -|       C  C  AAUAAC   UA    -   GU
  ACUCUCCCUC AAGGCUUC AA GC      CAGC CGUA CAU  A
  UGAGAGGGGG UUCCGGAG UU CG      GUCG GCAU GUG  A
AG           U^       -  -  GAC---   --    U   UC
        80        70             60          50
```

miR-n77 ΔG = -98.20 kcal/mol

```
           10          20        30        40
--   AC-    GG  A     .-AU  C|    GCCAG  A      G
  GCG   CCCA  UC GGCGG    UG GAUCG     CG UCCCGG A
  CGU   GGGU  AG CCGCU    AC CUGGC     GC AGGGCU U
GA   GUC    A-  A     \ --  -^    AG---  C      C
.       210        200         60            50
```

```
     70             80          90         100
A--     -----     GA  C     .-G    C              GG
   GGCUG     UGUUG  AU GCCUG   CAGC AUGCC--GCCGGUU  \
   CCGGC     ACGGC  UA CGGAC   GUCG UACGG  CGGCUAA  A
GGA     UAAAA     UG  -     \ -    -     \        GA
       190       180            140              110
```

```
 120
A    AG
 GCGC  A
 CGUG  C
A    UU
130
```

```
      150       160
GAAAAAU    A    AG
       CGGC GCGC  A
       GCCG CGCG  G
-------    A    UU
       170
```

miR-n78 ΔG = -70.10 kcal/mol

```
            10        20        30        40         50        60        70
----|                 UU   C           AAAAUA    ACAC-     AUCC       GA   U
    UCUAACAACACCAAGAAA  UCA CAACAAUGAUC      GGGC     GAGCU    AAAGGUG  UAG A
    AGGUUGUUGUGGUUCUUU  AGU GUUGUUACUAG      CCCG     CUCGG    UUUCCAC  AUU G
UCUC^                 UU   A           CUUCA-    AUAUA     CGUA       UC   C
      140       130       120       110        100        90        80
```

miR-n79 ΔG = -29.20 kcal/mol

```
          10        20        30
CA--                     A  CCAC-| GUCAAA
    UUCAAUAGGCAAUGAUCUUAA CA     UG      A
    AGGUUAUCCGUUACUAGAAUU GU     AC      U
UCAG                     C  AAUUA^ AUCCCC
80        70        60        50        40
```

miR-n80 ΔG = -49.20 kcal/mol

```
          10         20        30        40
--           -|            GAAA  U   U     A
  UGACAGAAGAG AGUGAGCACACAC    GC AAU GUAUG A
  ACUGUCUUCUC UCACUCGUGUGUG    CG UUA CAUAC A
CG           U^            GGA-  -   C     G
    80        70        60          50
```

miR-n81 ΔG = -41.20 kcal/mol

```
          10         20         30        40
U--          -          U  U  -|  A     UGGAG
   UGACAGAAGA UAGAGAGCAC GA GA UGA AUGCA     \
   ACUGUCUUCU AUCUCUCGUG UU CU ACU UACGU     C
ACU          G          U  C  C^  C     UAAUU
      80        70        60        50
```

miR-n82 ΔG = -61.90 kcal/mol

```
          10        20        30        40            50         60        70        80
--             C                    A    ACACCAA----     A  -|     AA  AAGAUUUGAA   AUUAUACAC
  UUCAAAUCUUCCU CCAAAACAUUGUUUUUGGUG GUGU           GAACA UG UUUUGG  GG          GUG          A
  AAGUUUAGAAGGA GGUUUUGUAACAAGAACCAC CACA           UUUGU AC AGAACC  CC          CAC          C
UG             A                    C    CCCACCCACAC     A  A^     A-  AACACCCACC   ACCCACACC
.       170       160       150       140       130       120        110       100        90
```

miR-n83 ΔG = -88.90 kcal/mol

```
        10        20        30        40        50        60        70        80        90       100       110
|         A             A                     CC                                    AACA   AAUAAAAAUU   A  A   C
AAUAGUUUCA AAUCAAUUUAAAU UACUUCAAAUAUUUUUUAUAA  UUUUUUAAUUAUUUAACAACUUAGAUAAAGUAUUU    ACC           GCA UA AUC \
UUAUCAAAGU UUAGUUAAAUUUA AUGAAGUUUAUAAAAAAUAUU  AAAAAGUUAAUAAAUUGUUGAAUCUAUUUUAUAAA    UGG           CGU AU UAG A
^         C             C                     AU                                    AAG-   AACUUUACAC   -  A   A
 .       210       200       190       180       170       160       150       140        130       120
```

miR-n84 ΔG = -80.40 kcal/mol

```
          10        20        30        40        50        60        70        80        90
GA--|    UU          CU      CG  C       UU      G  G   AC        U  AA      AGAAAUUAAGGG     UA
    GAGCU  CUUCAGUCCA  CAUGGA  GG GAAGGGU  GGAUUA CU CCG  UCAUUCAU CA  CACAGU            AGCAG  \
    CUCGA  GAAGUCAGGU  GUGUCU  UC UUUCCUA  UUUAAU GA GGC  AGUAAGUG GU  GUGUUA            UCGUC  U
CUCC^    GG          UC      CU  U       CU      A  G   GU        U  AA      ------------     GG
       170       160       150       140       130       120       110                   100
```

miR-n85 ΔG = -40.30 kcal/mol

```
          10        20          30        40        50
--     UU     U        --|      CUUUCUUU        UUUG   CCGAA
  UGCAU  GCACC GCACCUUA  CUUGUUU        UGUUUUAA    ACU     \
  ACGUG  CGUGG CGUGGAGU  GAACAAA        ACAAAGUU    UGA     A
GG     GC     U        UC^      AUU-----        UGAA   AAACC
110       100        90        80             70        60
```

miR-n86 ΔG = -73.70 kcal/mol

```
          10        20         30             40        50        60           70        80
--               A     UU-   UUUGCCA---    .-ACAAAUAUAUAAAGC|     UG-  UCCA--       U  UAAAAUGG--   A
  CCUCGCUCCCAGCUG CACCC   ACA          UGGC                 CUGCCU   UG      AAUCUGA UG          UGC A
  GGAGCGAGGGUUGAC GUGGG   UGU          GCCG                 GACGGA   AC      UUAGGCU AC          ACG C
CU               C     UUU   UUGAGUAGAG    \ ---------------^     UUG  UUCCUC       C  UUACUCGAGA   U
   190       180       170       160                       130       120       110       100        90

                                                     140
                                             U--     AGA
                                                GGCCU   \
                                                UCGGA   U
                                             AUG     ACU
                                             150
```

miR-n87 ΔG = -47.20 kcal/mol

```
           10          20         30
--      U            .-GA   -     C   UUA
  UCCAUA GUUCAUCAAUUU    GCU ACGAG GAG   \
  AGGUAU UAAGUAGUUAAA    UGA UGCUC CUC   A
UG      U            \ --   C     -   UGC
   150       140          50         40


                      60            70           80
                      GCC--   C| C  CAA----      .-AU     UC
                           UGC AU AU       ACUCCAU    GUUCA  A
                           ACG UA UG       UGAGGUA    CGAGU  A
                      AAACA   A^ U  ACUUCUG      \ --     UU
                        130       120       110         90


                                                        100
                                                   UAC    C
                                                      GAG G
                                                      CUU A
                                                   A--    G
```

miR-n88 ΔG = -53.30 kcal/mol

```
         10          20         30         40
A            -|            AAA   U    U   A
 UGACAGAAGAGA GUGAGCACACACG   GUA AUGG AUG A
 ACUGUCUUCUCU CACUCGUGUGUGC   CGU UACC UAC G
G            G^            GA-   -    U   G
    80         70         60           50
```

miR-n89 ΔG = -42.50 kcal/mol

```
          10        20        30        40
--|   A            U          CUCUC    U     U   A
  UUCC AAGGGAUCGCAU GAUCUAAUGA     GAUG CUAAA CAU U
  AAGG UUCCCUAGCGUA CUAGGUUGCU     UUAU GAUUU GUA U
UU^   G            -          -----    U     -   A
  90        80         70             60         50
```

miR-n90 ΔG = -34.10 kcal/mol

```
              10         20          30
------         A-     C     U-    -|   UU
      UAUGGGAGG  UUGGA AGGAC  GCUU GGUU  A
      AUACCCUCC  AACCU UCUUG  UGAA CCAA  A
GUGGCC         GC     U     UC    G^   UU
    70        60        50        40
```

miR-n91 ΔG = -47.70 kcal/mol

```
          10        20         30        40
--|         C    U     UU-   AAC   U    AUA    -  GG
  UAGCCAAGGA GAUU GCCUG   CCU   AAU UGGG   UGCC CU  A
  AUCGGUUCCU CUGA CGGAC   GGG   UUA ACCC   ACGG GA  C
GG^         -    -     UUU   CC-   C    AGG    A  AU
        90          80         70        60        50
```

miR-n92 ΔG = -49.60 kcal/mol

```
          10        20        30        40             50
--      U     C    G       CCAC     CCGAG   -    ----|  GC
  AACCCC AGUUG ACGU GACGUGU    GCCCA     GGG UUGG    GGC  \
  UUGGGG UCAAU UGUA CUGCGCA    UGGGU     CCC GACC    CCG  G
GG      U     C    A       AUUA     UGAA-   A    CAUA^  AU
       100        90        80        70         60
```

miR-n93 ΔG = -30.40 kcal/mol

```
          10        20
--|            UAC        AC
  AAGAUUUGGCCCG   CGAACCGA  U
  UUCUAAACUGGGU   GCUUGGCU  G
CU^            CUA        CG
     50        40        30
```

miR-n94 ΔG = -38.30 kcal/mol

```
          10         20             30
--  A        -   U       GG   GC-----|   U
  GG GCGACCUG GAA CACAUGU  GCU       ACCC C
  CC CGCUGGAC CUU GUGUACA  CGG       UGGG C
GU  C        U   -       A-   GUUCUCU^   U
     70        60          50        40
```
