# Supplementary figures and images for: Genome-Wide Analysis of MicroRNA Responses to the Phytohormone Abscisic Acid in Populus euphratica
Source: Front Plant Sci. 2016 Aug 17;7:1184. doi: 10.3389/fpls.2016.01184 (PMC4988358; doi:10.3389/fpls.2016.01184)

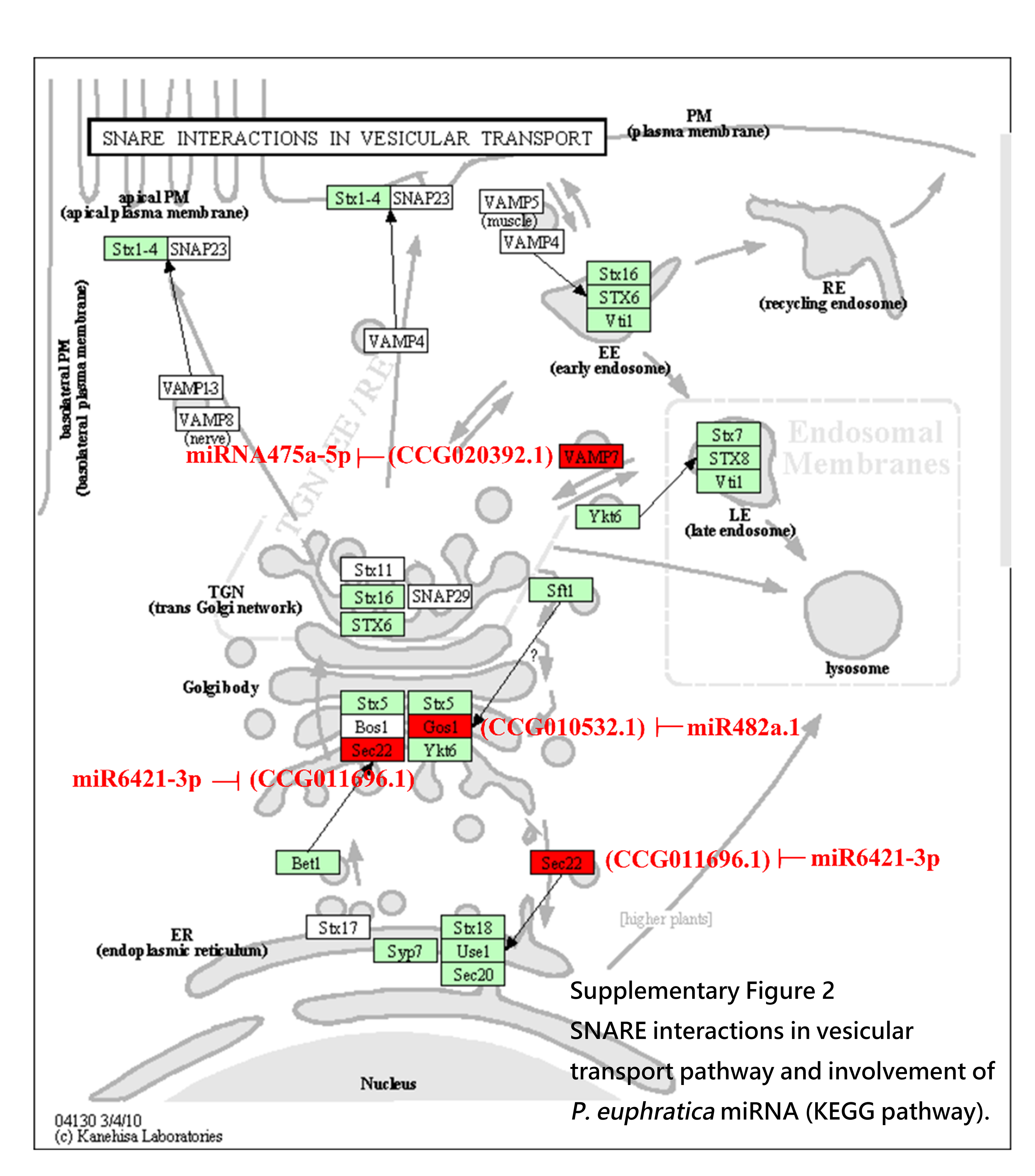

Supplement: Supplementary file 9 [file Image2.tif]
